# Synthetic zwitterions as efficient non-permeable cryoprotectants

**DOI:** 10.1038/s42004-021-00588-x

**Published:** 2021-10-28

**Authors:** Yui Kato, Takuya Uto, Daisuke Tanaka, Kojiro Ishibashi, Akiko Kobayashi, Masaharu Hazawa, Richard W. Wong, Kazuaki Ninomiya, Kenji Takahashi, Eishu Hirata, Kosuke Kuroda

**Affiliations:** 1 Faculty of Biological Science and Technology, Institute of Science and Engineering, Kanazawa University, Kakuma-machi, Kanazawa, 920-1192 Japan; 2 Organization for Promotion of Tenure Track, University of Miyazaki, Nishi 1-1 Gakuen-Kibanadai, Miyazaki, 889-2192 Japan; 3 Genetic Resources Center, National Agriculture and Food Research Organization, Kannondai, Tsukuba, 305-8602 Japan; 4 Cancer Research Institute, Kanazawa University, Kakuma-machi, Kanazawa, 920-1192 Japan; 5 Cell-Bionomics Research Unit, Institute for Frontier Science Initiative, Kanazawa, Ishikawa 920-1192 Japan; 6 WPI-Nano Life Science Institute, Kanazawa University, Kanazawa, Kanazawa, Ishikawa 920-1192 Japan; 7 Institute for Frontier Science Initiative, Kanazawa University, Kakuma-machi, Kanazawa, 920-1192 Japan; 8 NanoMaterials Research Institute, Kanazawa University, Kakuma-machi, Kanazawa, 920-1192 Japan

**Keywords:** Biophysical chemistry, Biomaterials - cells

## Abstract

Cryopreservation of cells is necessary for long periods of storage. However, some cell lines cannot be efficiently cryopreserved, even when optimized commercial cryoprotectants are employed. Previously, we found that a low-toxic synthetic zwitterion aqueous solution enabled good cryopreservation. However, this zwitterion solution could not cryopreserve some cells, such as human kidney BOSC cells, with good efficiency. Therefore, details of the cryoprotective effect of the zwitterions and optimization based on its mechanisms are required. Herein, we synthesized 18 zwitterion species and assessed the effects of the physical properties of water/zwitterion mixtures. Non-cell-permeable zwitterions can inhibit ice crystal formation extracellularly via direct interaction with water and intracellularly via dehydration of cells. However, cells that could not be cryopreserved by zwitterions were insufficiently dehydrated in the zwitterion solution. Dimethyl sulfoxide (DMSO) was combined as a cell-permeable cryoprotectant to compensate for the shortcomings of non-cell-permeable zwitterions. The water/zwitterion/DMSO (90/10/15, v/w/w) could cryopreserve different cells, for example freezing-vulnerable K562 and OVMANA cells; yielding ~1.8-fold cell viability compared to the case using a commercial cryoprotectant. Furthermore, molecular dynamics simulation indicated that the zwitterions protected the cell membrane from the collapse induced by DMSO.

## Abstract (web summary)

Zwitterionic solutions have potential applications as cryoprotectants, but the relationship between structure and performance is underexplored. Here a family of zwitterions are assessed in terms of cytotoxicity, cryopreservation performance, and effect on cell volume and water content.

## Introduction

Cryopreservation of cells is necessary for long storage periods to avoid mutations that might ensue during cultivation. It has enabled cell banking^1^. For example, >3600 cells are currently cryopreserved in the American Type Culture Collection^2^. When cells are cryopreserved in culture media, cryoprotectants are necessary because ice crystals form intra- and intercellularly and damage cells under extremely low temperatures^1,3–8^. A typical freezing medium is composed of medium/fetal bovine serum (FBS)/dimethyl sulfoxide (DMSO) (70/20/10, v/v/v). DMSO is cell-permeable^9^ and inhibits the formation of ice crystals intra- and extracellularly by interacting with water molecules^10^. However, some cell lines cannot be efficiently cryopreserved even when optimized commercial cryoprotectants are employed. Therefore, novel, efficient cryoprotectants are in high demand. However, cryoprotectants have a long research history, and their optimization has been attempted for decades without success for freezing-vulnerable cells. To break this unsuccessful cycle, the discovery of compounds that have not been applied to cryopreservation is needed.

**Fig. 1:**
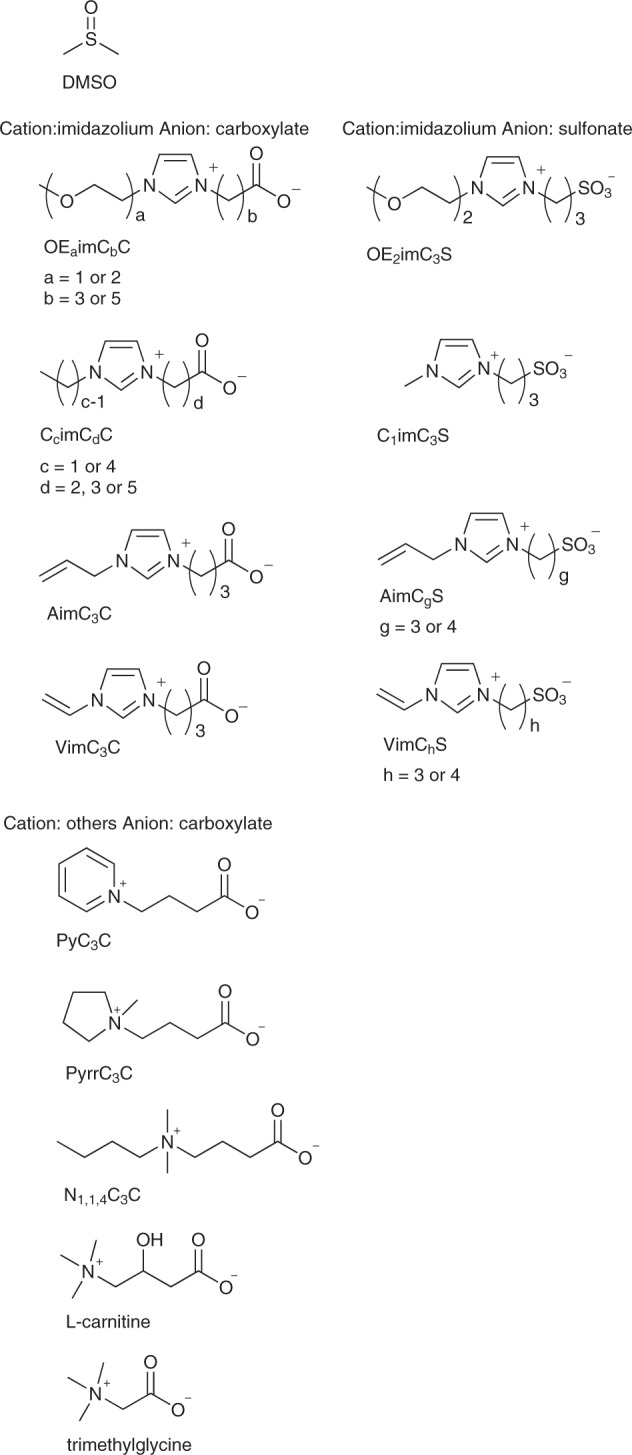
Structures of DMSO and the zwitterions used in this study. They are categorized by the cation and anion species.

Recently, we have developed a novel polar solvent, liquid zwitterion called OE_2_imC_3_C (Fig. 1), which exerts low toxicity to cells^11–13^. Unlike amino acids, OE_2_imC_3_C has electric charges, as it is aprotic. This charged molecule strongly interacts with water molecules and is expected to more strongly inhibit ice crystal formation than DMSO, possessing partial charges. One of the indicators of the strength of interaction with water is hydrogen bond basicity, which is represented by the *β* value of the Kamlet-Taft parameters. The *β* values of OE_2_imC_3_C and DMSO are 1.12 and 0.76, respectively^11,12^. As a result, we recently demonstrated the potential of OE_2_imC_3_C as a new type of cryoprotectant^13^. Water/OE_2_imC_3_C (95/5, v/w) cryopreserved human normal fibroblasts (hNF) and five other cell lines, as well as a DMSO-containing commercial cryoprotectant. The cryoprotective effect of OE_2_imC_3_C remains unclear, as it is a newly developed compound. In the present study, we carried out a detailed assessment of the limitations and potential of zwitterionic cryoprotectants, including OE_2_imC_3_C, and performed optimizations by analyzing the cryoprotective mechanisms employed. We revealed that cell dehydration was found to be a key factor for efficient cryopreservation. The mixtures of non-cell-permeable zwitterions and cell-permeable DMSO was found to cryopreserve freezing-vulnerable cells.

## Results and discussion

### Cryopreservation of cell lines with water/OE_2_imC_3_C (95/5, v/w)

Freezing media are typically prepared with concentrations based on volume/volume using pipettes; however, OE_2_imC_3_C aqueous solutions (OE_2_imC_3_C aqs.) are difficult to prepare at volume/volume due to their high viscosity. As a result, they were prepared at volume/weight in the present study. To investigate whether water/OE_2_imC_3_C (95/5, v/w) can be used as a universal cryoprotectant, nine cell lines including the cells cryopreserved in the previous study, were also cryopreserved, and the relative number of living cells after thawing was counted (Fig. S1). The cryoprotective effect was found to be significantly different depending on the cell line. For example, hNFs had the highest relative number of living cells of 1.10 while the number of living cells after cryopreservation with the commercial cryoprotectant was 1.00. In contrast, the human kidney cell line (BOSC) had the lowest number (0.20). These results indicate that the differences may not be based on the parental mammal species. Here, hNF, mNF, and BOSC were subjected to further studies as good, intermediate, and poor representatives, respectively.

### Optimization of the OE_2_imC_3_C-based freezing media

**Fig. 2:**
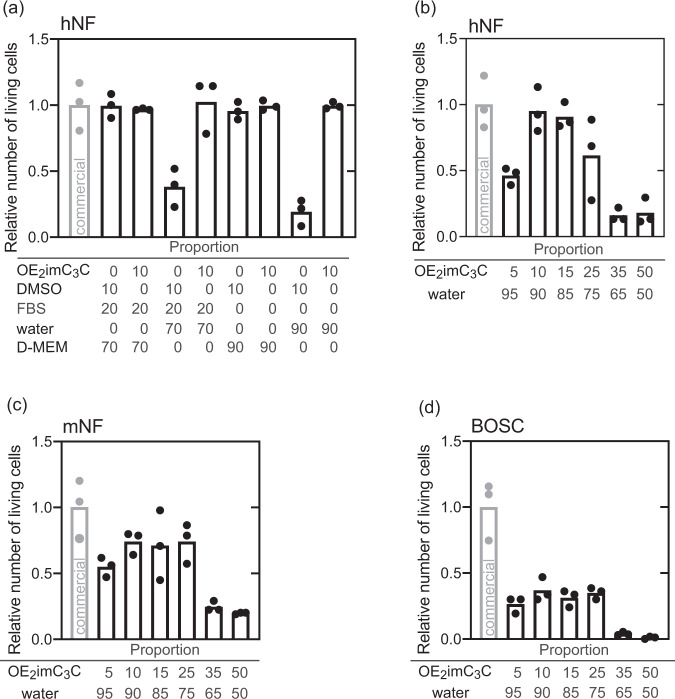
Cryopreservation using OE_2_imC_3_C-based freezing media. Relative number of living **a**, **b** hNF, **c** mNF, and **d** BOSC cells after cryopreservation with the indicated freezing media (*n* = 3, experimentally triplicate). In this experiment, DMSO was measured in volume. The commercial cryoprotectant employed was Culture Sure freezing medium (Fujifilm Wako Pure Chemical Corporation).

As shown above, we used a simple mixture containing OE_2_imC_3_C and water. However, based on the composition of the typical freezing medium (medium/FBS/DMSO; 70/20/10, v/v/v), we may have oversimplified this composition. Here, we carefully evaluated the components that are critical to achieve high cryopreserving efficiency relative to medium/FBS/DMSO. The relative numbers of living hNF cells after cryopreservation using medium/FBS/DMSO (70/20/10, v/v/v) and medium/FBS/OE_2_imC_3_C (70/20/10, v/v/w) were similar (0.99 and 0.97, respectively) (Fig. 2a). Such finding implies that OE_2_imC_3_C can act as an alternative to DMSO. However, the medium components critically affected the DMSO-based freezing media but not the OE_2_imC_3_C-based freezing media. This finding indicates that the cryopreservation mechanisms differ between DMSO- and OE_2_imC_3_C-based freezing media. DMSO is known as a cell-permeable cryoprotectant; however, non-cell-permeable components in media, such as NaCl, are required because the cell-permeable DMSO cannot regulate osmotic pressure, resulting in significant cell expansion (Fig. S2a). On the other hand, we reported that OE_2_imC_3_C hardly penetrates cells^13^. Water/OE_2_imC_3_C (90/10, v/w) has sufficient osmotic pressure (626 mOsm/kg) relative to the medium (Dulbecco’s modified Eagle’s medium with high glucose, 328 mOsm/kg), and the cells were not found to expand in the solution (Fig. S2a). Conversely, cell shrinkage was observed due to dehydration and will be discussed later. FBS did not affect the cryoprotection efficiency in either type of freezing medium. Similar trends were found in the mNF and BOSC (Fig. S3). Based on careful evaluation, we conclude that the simple freezing media composed of water and OE_2_imC_3_C were sufficient to subject to the next experiments.

We investigated the optimum concentration of OE_2_imC_3_C aqs. By determining the relative numbers of living hNF cells after cryopreservation using water/OE_2_imC_3_C (95/5–50/50, v/w), we found that water/OE_2_imC_3_C (90/10 and 85/15, v/w) was the optimal concentration (Fig. 2b). Because OE_2_imC_3_C is a non-cell-permeable cryoprotectant, dehydration of cells in water/OE_2_imC_3_C (95/5, v/w) may be insufficient to prevent intracellular ice crystal formation (Fig. S2a). In the cases of water/OE_2_imC_3_C (65/35, v/w) or higher concentration, the relative number of living cells after cryopreservation decreased as OE_2_imC_3_C might be toxic at such high concentrations. Rapid afflux and efflux of water during freezing and recovery are also known to induce damage to cells^1^ and they may also be involved. The toxicity of water/OE_2_imC_3_C (75/25, v/w), despite its very high concentration, is similar to that of the commercial cryoprotectant (Fig. S2c). The osmotic pressure, which is an indicator of cell dehydration, of water/OE_2_imC_3_C was found to be linearly increased (381, 626, 921, and 1501 mOsm/kg for 95/5, 90/10, 85/15, and 75/25 (w/v), respectively). Cell volume and the reciprocal of the osmotic pressure of the media were reported to display a linear relationship^1,14^. Herein, the volume of hNF decreased linearly. Similar trends were observed in the cases of mNF and BOSC (Fig. 2c, d) when mNF and BOSC were cryopreserved using water/OE_2_imC_3_C (95/5–50/50, v/w). Although relative numbers of living cells using OE_2_imC_3_C aqs. were lower than those using the commercial cryoprotectant, water/OE_2_imC_3_C (90/10–75/25, v/w) was identified as the optimal concentration range. Therefore, water/OE_2_imC_3_C (90/10 and 85/15, v/w) was identified as the universal optimal concentration range.

Notably, the relative number of living hNF cells after cryopreservation using water/OE_2_imC_3_C (95/5, v/w) is significantly different from that observed in the previous study (Fig. S1 vs. the present study Fig. 2b; 1.10 vs 0.46). This is at least partly based on the difference in cell counting methods: an automated cell counter (^®^Countess II FL, Thermo Fisher Scientific) and a hemocytometer in the previous and present studies, respectively. The living cell numbers counted by hemocytometers were lower than those counted by ^®^Countess II FL (Fig. S4). ^®^Countess II FL is known to show different cell numbers relative to hemocytometers^15^ and is not optimized for counting cells that are partly damaged by cryopreservation. Therefore, we opted to use hemocytometers for subsequent experiments.

### Assessment of cell line dependence on cryopreservation with OE_2_imC_3_C-based freezing media

**Fig. 3:**
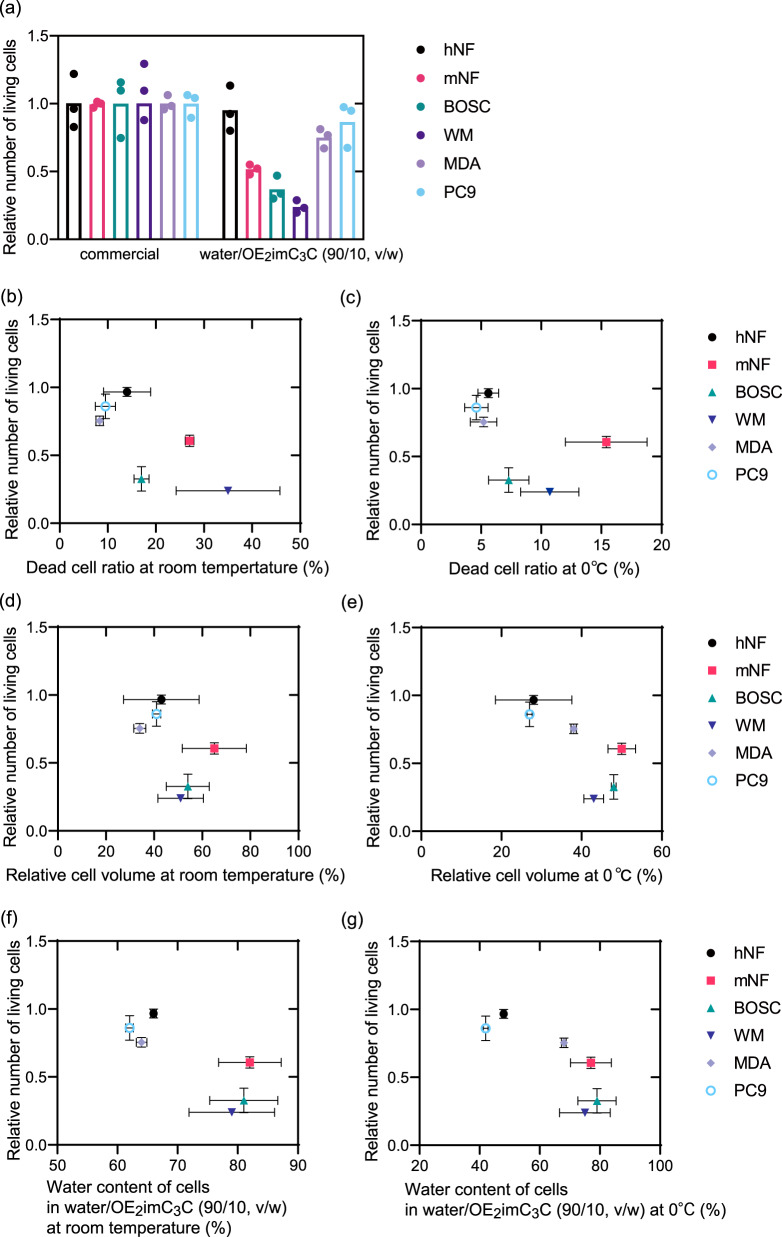
Cell behaviors in water/OE_2_imC_3_C (90/10, v/w). **a** Relative number of living cells after cryopreservation with water/OE_2_imC_3_C (90/10, v/w) (*n* = 3, experimentally triplicate). **b**, **c** Relation between relative number of living hNF, mNF, BOSC, WM, MDA, and PC9 cells and dead cell ratio at **b** room temperature and **c** 0 °C after 60 min of immersion in the indicated solution (*n* = 3, biologically triplicate). **d**, **e** Relation between a relative number of living hNF, mNF, BOSC, WM, MDA, and PC9 cells and relative cell volumes at **d** room temperature and **e** 0 °C after 5 min of immersion in the indicated solutions (cell volumes at the same temperature in PBS were standardized as 100%). **f**, **g** Relation between that the water contents of cells in water/OE_2_imC_3_C (90/10, v/w) at **f** room temperature and **g** 0 °C and the relative numbers of living hNF, mNF, BOSC, WM, MDA, and PC9 cells. (*n* = 3, biologically triplicate). **b**–**e** These cells were immersed as floating cells in the solutions after trypsinization. The bars show standard error.

Here, we cryopreserved hNF, mNF, BOSC, WM, MDA, and PC9 with the optimized water/OE_2_imC_3_C (90/10, v/w) (Fig. 3a). Even at optimal concentrations, the relative numbers of living cells after cryopreservation were considerably different according to the cell lines. Therefore, we examined the different behaviors of cell lines during cryopreservation.

The toxicity of OE_2_imC_3_C on the cell lines was investigated (Fig. 3b, c). Single dispersed cells were immersed in water/OE_2_imC_3_C (90/10, v/w) for 60 min, and the dead cell ratio was measured. Although all cells had higher dead cell ratios in water/OE_2_imC_3_C (90/10, v/w) than in PBS (Fig. S5a), the maximum dead cell ratio was 35% for WM at room temperature and 16% for mNF at 0 °C; this was insufficient to be directly linked to the low relative number of living cells after cryopreservation (<0.3 for WM). However, the well-cryopreserved cell lines (hNF, MDA, and PC9) showed relatively lower dead cell ratios than poorly cryopreserved cell lines (mNF, BOSC, and WM). Therefore, poorly cryopreserved cell lines might be vulnerable to exposure to OE_2_imC_3_C. Further discussions are however required before any conclusion can be drawn. For example, poorly cryopreserved cell lines showed high dead cell ratios even in PBS (Fig. S5a). It implies that poorly cryopreserved cell lines were very weak at floating dispersed state.

Non-cell-permeable cryoprotectants, such as OE_2_imC_3_C, can inhibit extracellular ice crystal formation via direct interaction with water and intracellular ice crystal formation via cell dehydration. According to the literature, it is more important to inhibit intracellular ice crystal formation than extracellular crystals^1,16^. To investigate dehydration, single dispersed cells were immersed in water/OE_2_imC_3_C (90/10, v/w), and the cell volumes were measured relative to those in PBS (Fig. 3d, e). All cells shrank in water/OE_2_imC_3_C (90/10, v/w). In particular, the well-cryopreserved cell lines (hNF, MDA, and PC9) showed significant shrinkage compared with the poorly cryopreserved cell lines (mNF, BOSC, and WM). These different dehydration behaviors were not based on their original cell sizes (Fig. S5b–e). Finally, we found a clear relation between that the water contents of cells in water/OE_2_imC_3_C (90/10, v/w) and the relative numbers of living cells (Fig. 3f, g and Fig. S5f–i). Therefore, the low cryoprotection efficiency was owing to insufficient cell dehydration, leading to the formation of intracellular ice crystals, as well as toxicity. The insufficient cell dehydration implies that the poorly cryopreserved cell lines contain much amount of low-molecular compounds such as ions. Collectively, these findings highlight the difficulty of achieving sufficient dehydration of all cells using OE_2_imC_3_C.

### Optimization of zwitterion-based freezing media and an in-depth discussion of the cryopreservation mechanism

**Fig. 4:**
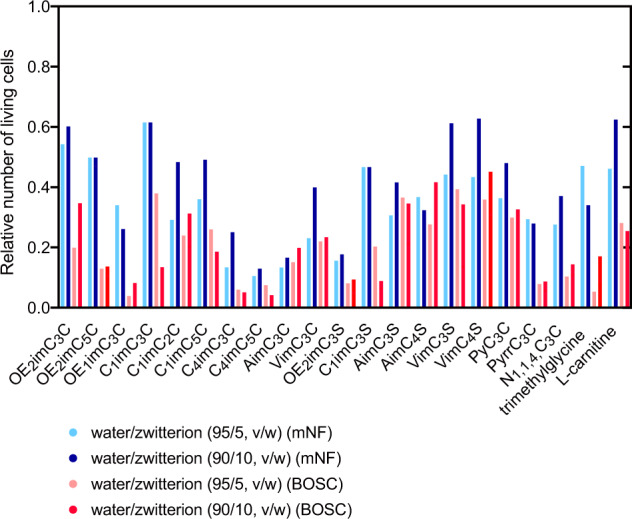
Relative number of living mNF and BOSC cells after cryopreservation using water/zwitterion (95/5 and 90/10, v/w). (*n* = 3, experimentally triplicate) The actual data points are shown in Fig. S6.

mNF and BOSC were subjected to cryopreservation using different water/aprotic zwitterion (95/5 and 90/10, v/w) (see Fig. 1 for all structures); this is because other aprotic zwitterions also can strongly interact with water owing to their charges. The cryoprotective effect was found to significantly differ based on the zwitterion species (Fig. 4). The findings indicate that the relative number of living cells after cryopreservation depends on both the zwitterion species and the cell lines. Nevertheless, a clear association between the cryoprotective effect and ionic structures was not found.

Similar non-zwitterionic-type ions, namely free ions called ionic liquids, are known to be toxic^11,17–19^; however, these ions were subjected to cryopreservation. Surprisingly, the toxic ionic liquid^13^ also exerted a cryopreservation effect at a certain degree (Fig. S7), indicating that toxicity at room temperature might not be related to the cryopreservation efficiency. This finding also implies that toxic compounds can be candidates for cryoprotectants.

The relationship between the osmotic pressures and the molar concentrations of zwitterions in the water/zwitterion (90/10, v/w) was plotted (Fig. S8) to discuss the cryopreservation mechanisms. As a result, a linear relationship was found: the osmotic pressures were related to the concentration but not the ion structure. The relationship between the osmotic pressures and the relative number of living cells (mNF and BOSC) was plotted (Fig. S9); however, no clear relation was found and osmotic pressure was not identified as a dominant factor for cryoprotection.

The physical state, especially glass transition, of water/zwitterion (90/10, v/w) at low temperature was examined using differential scanning calorimetry (DSC); this is because many successful examples based on glass transition have been reported in the case of fast cooling^20^. Glass transition is related to viscosity^1^. In fact, zwitterion solutions have high viscosity due to strong electrostatic interactions and are easy to transform into the glass state^11,21^. All zwitterion aqs. (90/10, v/w), except for VimC_4_S, showed glass transition (Table S1). Glass transition of zwitterion aqs. occurred at −75 to −105  °C. Here, cryopreservation was performed at −85 °C, and therefore, the glass transition temperatures coincidentally existed above and below the temperature. However, no clear relationship was found between the relative number of living cells and the glass transition temperatures (Fig. S10). Furthermore, the relative numbers of living cells were high (mNF and BOSC: 0.63 and 0.45, respectively), even when VimC_4_S aq. (90/10, v/w) which did not show glass transition was employed. Such findings suggest that the glass transition itself did not critically affect the cryoprotection effect.

The proportion of unfrozen water of zwitterion aqs. (90/10, v/w) at low temperatures was investigated. Small zwitterions were found to display a higher ratio due to their higher molar concentration. Sulfonate-type zwitterions showed a higher ratio than carboxylate-type zwitterions. An evident tendency was not observed between cell viability and the proportion of unfrozen water (Fig. S11). Therefore, unfrozen water might be necessary for protecting cells from extracellular ice crystals; however, excess amounts of unfrozen water may not exert further cryoprotective effects. Such findings suggest that the species and concentration of zwitterion significantly affect cell viability after cryopreservation; however, glass transition, glass transition temperature, osmotic pressure, and proportion of unfrozen water were not identified as the dominant factors for efficient cryopreservation. Therefore, the critical factors for efficient cryoprotection by zwitterion aqs. are presently under investigation. We also attempted cryopreservation with zwitterion/zwitterion mixtures; however, the blend did not exert synergistic and efficient cryopreservation (Fig. S12).

### Optimization via mixing with cell-permeable cryoprotectants

Herein, we reconfirmed the important ability of zwitterions to act as efficient non-cell-permeable cryoprotectants to inhibit extracellular ice formation. The proportion of unfrozen water in the 0.6 mol/L OE_2_imC_3_C aq. (water/OE_2_imC_3_C (85/15, v/w)) was 31 wt% at subzero temperature, whereas 11 wt% of the unfrozen water for equimolar sucrose aq., which is a typical non-cell-permeable cryoprotectant. Equimolar DMSO and glycerol aqs., which are typical cell-permeable cryoprotectants with the strong inhibitory ability for extra- and intracellular ice formation, showed 14 and 15 wt% of unfrozen water, respectively. These results clearly suggest that zwitterions are promising non-cell-permeable cryoprotectants.

**Fig. 5:**
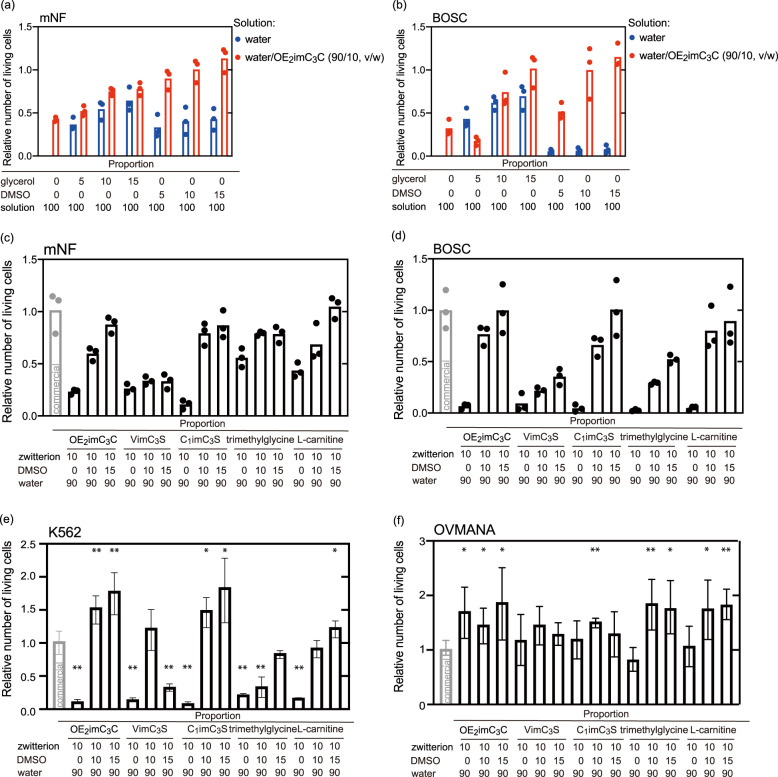
Cryopreservation of different cells using freezing media containing OE_2_imC_3_C and cell-permeable cryoprotectants. Relative number of living **a** mNF and **b** BOSC cells after cryopreservation with mixtures of water/OE_2_imC_3_C (90/10, v/w) and cell-permeable additives (glycerol, DMSO) (*n* = 3, experimentally triplicate). A relative number of living **c** mNF, **d** BOSC (*n* = 3, experimentally triplicate), **e** K562, and **f** OVMANA (*n* = 3, biologically triplicate) cells after cryopreservation with mixtures of water/zwitterion (90/10, v/w) and DMSO. (*p < 0.1, **p < 0.05, compared with commercial) The bars show standard error. Total compositions in some cases are over 100 to be clear. The commercial cryoprotectant employed was Culture Sure freezing medium (Fujifilm Wako Pure Chemical Corporation).

Blending zwitterions, efficient non-cell-permeating cryoprotectants, and cell-permeating cryoprotectants are expected to exert efficient cryopreservation. DMSO and glycerol were added to water/OE_2_imC_3_C (90/10, v/w) as additives. As expected, the mixtures resulted in higher relative numbers of living cells (Fig. 5a, b). Water/OE_2_imC_3_C/DMSO (90/10/15, v/w/w) was identified as the best combination while the relative numbers of living mNF and BOSC cells were 1.13 and 1.14, respectively; these numbers were respectively higher than those of cells cryopreserved with the commercial cryoprotectant. Glycerol is known to be less permeable and highly toxic relative to DMSO^1^ aligning with the low relative number of living cells. Adding typical non-cell-permeable cryoprotectants, sucrose, and FBS, into water/OE_2_imC_3_C (90/10, v/w) did not critically affect the relative number of living cells (Fig. S13), indicating that blending with cell-permeable cryoprotectants is important.

Mixtures of different zwitterions (OE_2_imC_3_C, VimC_3_S, C_1_imC_3_S, trimethylglycine, l-carnitine) and DMSO were employed. Except for VimC_3_S-based freezing media, these mixtures cryopreserved mNF and BOSC in a degree similar to the commercial cryoprotectant. For instance, water/C_1_imC_3_S/DMSO (90/10/15, v/w/w) led to relative numbers of living mNF and BOSC of 0.92 and 1.01, respectively. Although OE_2_imC_3_C and VimC_3_S without any additives showed similar cryoprotecting effects (see Fig. 4), their cryoprotective effect differed when combined with DMSO; DMSO significantly improved OE_2_imC_3_C-based media but not VimC_3_S-based media. Such findings suggest that the cryoprotective effect of the zwitterion itself is not related to that of mixtures containing DMSO.

Six cell lines presented in Fig.  S1 and two cell lines (Vn1919 and HL-60) were cryopreserved using water/zwitterion/DMSO to confirm the universality of the mixtures (Fig. S14). The relative numbers of living cells were found to be improved, including the relative numbers of the cell lines that were poorly cryopreserved by water/zwitterions, such as B16F10 and 4T1. The results suggested that the water/OE_2_imC_3_C/DMSO (90/10/15, v/w/w) and water/C_1_imC_3_S/DMSO (90/10/15, v/w/w) were universal cryoprotectants.

The cell lines mentioned above are cryopreserved by the commercial cryoprotectant with high viability, and the high efficiency of water/zwitterion/DMSO is difficult to demonstrate. Here, we cryopreserved two additional cell lines (K562 and OVMANA) that are vulnerable to freezing. Water/zwitterion/DMSO resulted in higher relative numbers of living cells than did the commercial cryoprotectant (Fig. 5e–f). In particular, water/OE_2_imC_3_C/DMSO and water/C_1_imC_3_S/DMSO (90/10/15, v/w/w) cryopreserved K562 cells (relative number of living cells: 1.74 and 1.80, respectively). Moreover, both water/zwitterion and water/zwitterion/DMSO cryopreserved OVMANA well. Cell proliferation following cryopreservation with water/OE_2_imC_3_C/DMSO (90/10/15, v/w/w) and water/C_1_imC_3_S/DMSO (90/10/15, v/w/w) was found to be equivalent to that with the commercial cryoprotectant (Fig. S15a, b). The water/zwitterion/DMSO mixtures were thus identified as promising freezing media for cell lines or cells that are vulnerable to freezing. As these mixtures consist of only three components, additional inclusions could be made to improve their effects, as commercial cryoprotectants are optimized by blending several additives to adjust pH and osmotic pressure.

### Cryoprotecting mechanisms of water/OE_2_imC_3_C/DMSO

**Fig. 6:**
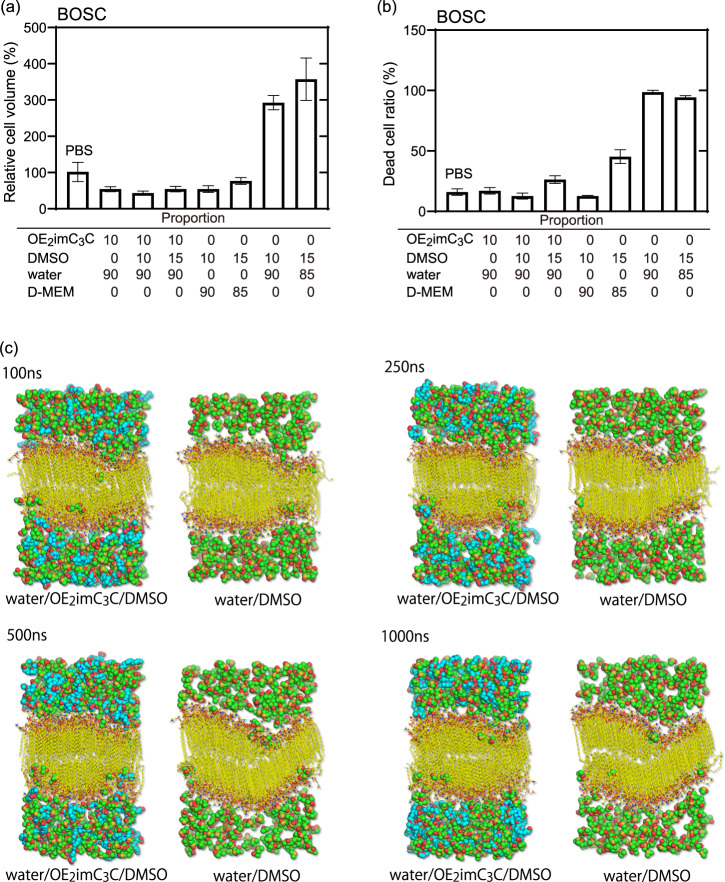
Cryoprotecting mechanisms of zwitterion/DMSO-containing freezing media. **a** Relative BOSC cell volume after 5 min of immersion in the indicated solutions (cell volume in PBS was standardized as 100%). Total compositions in some cases are over 100 to be clear. **b** Dead BOSC cell ratio after 60 min of immersion in the indicated solution (*n* = 3, biologically triplicate). **a** and **b** The cells were immersed as floating cells in the solutions at room temperature after trypsinization. The bars show standard error. **c** Images of the cell membrane in the water/OE_2_imC_3_C/DMSO and water/DMSO mixtures at 100, 250, 500, and 1000 ns, as calculated by molecular dynamics simulations with Lipid17 force field. The reproducibility was confirmed by the calculation with Lipid14 force field (Fig. S27).

OE_2_imC_3_C is a non-cell-permeable cryoprotectant that strongly inhibits extracellular ice formation, while DMSO is a cell-permeable cryoprotectant that inhibits intracellular ice formation. To determine the synergistic effect of water/OE_2_imC_3_C/DMSO, cell volume was measured (Fig. 6a). The water/DMSO mixtures had large cell volumes, suggesting that DMSO requires non-cell-permeable cryoprotectants, such as OE_2_imC_3_C or NaCl (contained in culture media). Water/OE_2_imC_3_C/DMSO (90/10/15, v/w/w) exhibited the same cell volume as water/OE_2_imC_3_C (90/10, v/w). This dehydration inhibited intracellular ice formation. In addition, because DMSO exists in cells, the water content of cells was assumed to be lower in water/OE_2_imC_3_C/DMSO than in water/OE_2_imC_3_C. By estimating the toxicity of the freezing media, the water/DMSO mixtures were highly toxic and were associated with cell expansion, as mentioned above. Interestingly, the toxicity of water/OE_2_imC_3_C/DMSO (90/10/15, v/w/w) was lower than that of medium/DMSO (85/15, v/w) (Fig. 6b). Accordingly, OE_2_imC_3_C was thought to reduce the toxicity of DMSO. Because water efflux by high osmotic pressure occurs more quickly than DMSO influx, OE_2_imC_3_C may suppress the influx of DMSO.

Based on previous studies, some cryoprotectants can stabilize cell membranes^22,23^. Accordingly, a molecular dynamics simulation was conducted to assess the stabilization of the cell membrane (Fig. 6c). In DMSO/water, the cell membrane structure was found to collapse and was partly converted into an interdigitated phase within 250 ns. However, in water/OE_2_imC_3_C/DMSO, the cell membrane did not critically collapse, even at 1000 ns, despite a certain degree of disruption. The collapse of the lipid membrane was quantitatively assessed by electron density and surface area (Figs. S16 and S17). The electron density of DPPC lipid molecules in the water/DMSO changed from the initial one around the center of the membrane. It indicates that the lipid membrane was transformed into a partly interdigitated structure. On the other hand, the lipid membrane in the water/OE_2_imC_3_C/DMSO relatively maintained the initial electron density.

The penetration of DMSO molecules can disrupt the organized structure of the lipid bilayer. It was observed that the penetration behavior of DMSO molecules to the lipid bilayer shifted in the presence of OE_2_imC_3_C (Fig. S18). The number density of DMSO molecules at the center of the membrane was 1.42 × 10^−7^ and 3.44 × 10^−7^/Å^3^ in the water/OE_2_imC_3_C/DMSO and water/DMSO solutions, respectively. The results may be caused by interaction between DMSO and OE_2_imC_3_C, which is stronger than that between DMSO and DPPC lipid molecules, as shown in Figs. S19 and 20. The interaction between OE_2_imC_3_C and lipid molecules (Figs. S21–S23) can also contribute to shift in surface charge of cell membrane: we found a slight decrease in the DMSO/lipid interaction and no change in the DMSO/water interaction in the presence of OE_2_imC_3_C (Fig. S20, 24). From the discussion, OE_2_imC_3_C interacts with DMSO and lipid and then inhibits the membrane permeation of DMSO, resulting in stabilization of the membrane. This simulation supports one aspect of cryoprotection mechanisms and further study will be required to clarify.

We here suspect that OE_2_imC_3_C increased the viscosity, which might have affected the simulation results. However, for DMSO and water molecules, the mean square displacements, which are related to diffusivity, in water/OE_2_imC_3_C/DMSO was ~80% of that in DMSO/water (Fig. S25). Such findings indicate that viscosity is not a critical factor.

Furthermore, we also analyzed the interaction between OE_2_imC_3_C/water molecules as shown in Figs. S26. It was observed the water structuring near the anion part of OE_2_imC_3_C. Hence, it supports the mechanism for cryoprotecting effect: inhibition of ice crystal formation by OE_2_imC_3_C. Based on these results, OE_2_imC_3_C may be a promising cryoprotectant that shields cells from the adverse effects of DMSO. The cell lines (K562 and OVMANA) that are vulnerable to freezing could be effectively cryopreserved through the complex mechanisms.

## Conclusion

Herein, zwitterion aqs. were found to exert their cryoprotective effect via cell dehydration, inhibition of extracellular ice formation, and low cytotoxic effects. By altering the structures of the zwitterions, we investigated the impact of the physical properties of the water/zwitterion mixtures. The glass transition, glass transition temperature, osmotic pressure, and proportion of unfrozen water were not identified as the dominant factors for efficient cryopreservation. On the other hand, cell dehydration was found to be a key factor. Although zwitterions were found to be promising non-cell-permeable cryoprotectants, none of the water/zwitterion mixtures resulted in good cryopreservation efficiency for BOSC. As a result, DMSO was blended as a cell-permeable cryoprotectant to compensate for the shortcoming of the non-cell-permeable zwitterions. Water/OE_2_imC_3_C/DMSO or water/C_1_imC_3_S/DMSO (90/10/15, v/w/w) was found to cryopreserve different cells. In particular, K562 and OVMANA cells, which are vulnerable to freezing, were more efficiently cryopreserved with this mixture than with the commercial cryoprotectant. Altogether, OE_2_imC_3_C was found to strongly inhibit extracellular ice formation and protect cells from the adverse effects of DMSO, resulting in the high cryoprotecting efficiency when mixing with DMSO.

## Methods

### Materials

OE_2_imC_3_C, OE_2_imC_5_C, and C_1_imC_3_C were synthesized as previously reported^11^. OE_1_imC_3_C, C_1_imC_5_C, C_4_imC_3_C, C_4_imC_5_C, AimC_3_C, VimC_3_C PyC_3_C, PyrrC_3_C, and N_1,1,4,_C_3_C were synthesized using a previously reported method with minor modifications^11^. C_1_imC_3_S was synthesized as previously reported^24^. OE_2_imC_3_S, AimC_3_S, AimC_4_S VimC_3_S, and VimC_4_S were synthesized using a previously reported method with minor modifications^24^. C_1_imC_2_C was synthesized as previously reported^25^. The reagents used to synthesize the above-mentioned zwitterions (2-bromoethyl methyl ether, ethyl 6-bromohexanoate, 1-butylimidazole, 1-allylimidazole, 1-vinylimidazole, 2-bromoethyl methyl ether, 1,3-propenesultone, pyridine, pyrroridine, and dimethylbutylamine) were purchased from Tokyo Chemical Industry Co., Ltd., and used as received. Trimethylglycine, l-carnitine, and DMSO were purchased from Tokyo Chemical Industry Co., Ltd., and used as received. Glycerol and sucrose were purchased from Nacalai Tesque, Inc., and used as received.

### Cell

hNF, mouse normal fibroblast (mNF) derived from C57BL/6-EGFP mice, MDA-MB-231 human breast cancer cells (MDA), WM266.4 human melanoma cells (WM), B16F10 mouse melanoma cells, and 4T1 mouse breast cancer cells were kind gifts from professor Erik Sahai (The Francis-Crick Institute, UK). BOSC human kidney cells, PC9 human lung cancer cells, Mardin-darby canine kidney cells (MDCK) were kind gifts from professor Michiyuki Matsuda (Kyoto University), professor Seiji Yano (Cancer Research Institute of Kanazawa University), and professor Etsuko Kiyokawa (Kanazawa Medical University), respectively. HL-60 human promyelocytic leukemia cells were kindly gifted from professor Atsushi Hirano (Cancer Research Institute of Kanazawa University). K562 human chronic myelogenous leukemia cells and Vn1919 neuroglial and neuronal character co-expressing ependymoma cells were used in the previous studies^26,27^. OVMANA human ovarian tumor cells were purchased from the Japanese Collection of Research Bioresources.

### Cell culture

hNF, mNF, BOSC, WM, MDA, PC9, B16F10, 4T1, and Vn1919 were grown and maintained as monolayer cultures at 37 °C in 5% CO_2_ humidified atmosphere, using Dulbecco’s modified Eagle’s medium (high glucose with l-glutamine and phenol red, Fujifilm Wako Pure Chemical Corporation) supplemented with 1 vol% penicillin–streptomycin solution (×100) (Fujifilm Wako Pure Chemical Corporation) and 10 vol% FBS (Sigma-Aldrich Co., Llc.). HL-60 and K562 were grown and maintained as floating cultures at 37 °C in 5% CO_2_ humidified atmosphere, using Roswell Park Memorial Institute medium (RPMI, Nacalai Tesque, Inc.) supplemented with 1 vol% penicillin–streptomycin solution and 10 vol% FBS. OVMANA was grown and maintained as monolayer cultures at 37 °C in 5% CO_2_ humidified atmosphere, using RPMI, Nacalai Tesque, Inc. supplemented with 1 vol% penicillin–streptomycin solution and 10 vol% FBS. The cells were sub-cultured every 4–6 days with or without trypsin solution (0.5 w/v% trypsin-5.3 mmol/L EDTA・4Na solution without phenol red (×10), Fujifilm Wako Pure Chemical Corporation).

### Cryopreservation

The zwitterion and zwitterion/DMSO solutions were prepared via mixing with ultrapure water. Thereafter, cells (1 × 10^6^ cells) were collected and centrifuged (100 × *g*, 5 min at room temperature). After removing the supernatant, 100 μL of cryoprotectants were added and pipetted slowly. The samples were stored in a box (Mr. Frosty, Thermo Fisher Scientific Inc.) in a –85 °C freezer for 3–5 days. For sample thawing, a culture medium incubated at 37 °C was added to the frozen samples. The relative number of living cells was counted using a hemocytometer (Fukaekasei Corporation and Watson Corporation) with trypan blue (Fujifilm Wako Pure Chemical Corporation).

$${{{{{{{\mathrm{Relative}}}}}}}}\;{{{{{{{\mathrm{number}}}}}}}}\;{{{{{{{\mathrm{of}}}}}}}}\;{{{{{{{\mathrm{living}}}}}}}}\;{{{{{{{\mathrm{cells}}}}}}}} = \frac{{{{{{{{{\mathrm{Counted}}}}}}}}\;{{{{{{{\mathrm{living}}}}}}}}\;{{{{{{{\mathrm{cell}}}}}}}}\;{{{{{{{\mathrm{number}}}}}}}}\;({{{{{{{\mathrm{sample}}}}}}}})}}{{{{{{{{{\mathrm{Counted}}}}}}}}\;{{{{{{{\mathrm{living}}}}}}}}\;{{{{{{{\mathrm{cell}}}}}}}}\;{{{{{{{\mathrm{number}}}}}}}}\;({{{{{{{\mathrm{commercial}}}}}}}}\;{{{{{{{\mathrm{cryoprotectant}}}}}}}})}}$$

For the proliferation studies, K562 and OVMANA cells after cryopreservation were seeded in a six-well plate. K562 cells were counted sequentially. OVMANA cells were counted after trypsin treatment when the most-grown cells were at 80% confluent.

The commercial cryoprotectant employed was Culture Sure freezing medium (DMSO-containing, Fujifilm Wako Pure Chemical Corporation). This is one of the typical commercial cryoprotectants and is suitable for comparison in cryopreservation efficiency. In the present study, the relative number of living cells was employed because the absolute number of living cells was variable, based on biological variation, even when using the commercial cryoprotectant. The absolute numbers of living cells given by the commercial cryoprotectant were roughly associated with those given by the sample solutions. In addition, most experiments were experimentally triplicated in this study. The results therefore still contain a certain amount of error based on the biological variation but are sufficient to suggest rough trends and relations.

When preparing the freezing medium, the amount of culture medium, FBS, and water were measured by volume using pipettes. The number of zwitterions, DMSO, an ionic liquid, glycerol, and sucrose were measured by weight using an electronic balance unless we note.

### Toxicity of cryoprotectants

Cells (1 × 10^5^ cells) after trypsinization were collected and centrifuged (100 × *g*, 5 min at room temperature). Following the removal of the supernatant, 100 μL of cryoprotectants were added and pipetted slowly. The cells were incubated at room temperature (15–25 °C) and 0 °C as floating cells in cryoprotectants. After 60 min, the dead cell ratio was evaluated by counting using a hemocytometer and trypan blue.

$${{{{{{{\mathrm{Dead}}}}}}}}\;{{{{{{{\mathrm{cell}}}}}}}}\;{{{{{{{\mathrm{ratio}}}}}}}}\;({{{{{{{\mathrm{\% }}}}}}}}) = \frac{{{{{{{{{\mathrm{Number}}}}}}}}\;{{{{{{{\mathrm{of}}}}}}}}\;{{{{{{{\mathrm{dead}}}}}}}}\;{{{{{{{\mathrm{cells}}}}}}}}}}{{{{{{{{{\mathrm{Number}}}}}}}}\;{{{{{{{\mathrm{of}}}}}}}}\;{{{{{{{\mathrm{living}}}}}}}}\;{{{{{{{\mathrm{cells}}}}}}}} + {{{{{{{\mathrm{Number}}}}}}}}\;{{{{{{{\mathrm{of}}}}}}}}\;{{{{{{{\mathrm{dead}}}}}}}}\;{{{{{{{\mathrm{cells}}}}}}}}}}$$

### Cell volumes in cryoprotectants

Cells (1 × 10^5^ cells) after trypsinization were collected and centrifuged (100 × *g*, 5 min at room temperature). After removing the supernatant, 100 μL of cryoprotectants were added and pipetted slowly. Thereafter, the cells were incubated at room temperature (15–25 °C) or 0 °C as floating cells in cryoprotectants. After 5 min, images of the cells were captured with an optical IX83 inverted microscope (Olympus Corporation). The cell radii were measured using the software “ImageJ” (ImageJ 1.52p, Wayne Rasband, National Institutes of Health, USA). Relative cell volumes were calculated using the measured cell radii, relative to the cell volume in phosphate-buffered saline (PBS). Relative cell volume was defined as the following equation.

$${{{{{{{\mathrm{Relative}}}}}}}}\;{{{{{{{\mathrm{cell}}}}}}}}\;{{{{{{{\mathrm{volume}}}}}}}}\;(\% ) = \frac{{{{{{{{{\mathrm{Cell}}}}}}}}\;{{{{{{{\mathrm{volume}}}}}}}}\;{{{{{{{\mathrm{in}}}}}}}}\;{{{{{{{\mathrm{water}}}}}}}}/{{{{{{{\mathrm{OE}}}}}}}}_2{{{{{{{\mathrm{imC}}}}}}}}_3{{{{{{{\mathrm{C}}}}}}}}\;\left( {90/10,\;{{{{{{{\mathrm{v}}}}}}}}/{{{{{{{\mathrm{w}}}}}}}}} \right)\;\left( {\mu {{{{{{{\mathrm{m}}}}}}}}^3} \right)}}{{{{{{{{{\mathrm{Cell}}}}}}}}\;{{{{{{{\mathrm{volume}}}}}}}}\;{{{{{{{\mathrm{in}}}}}}}}\;{{{{{{{\mathrm{PBS}}}}}}}}\;\left( {\mu {{{{{{{\mathrm{m}}}}}}}}^3} \right)}} \times 100$$

### Water content of cells

Cells (1 × 10^7^ cells) after trypsinization were collected and centrifuged (100 × *g*, 5 min at room temperature). After removing the supernatant, the weight of the cells was measured. Thereafter, the cells were dried in vacuo (1 Pa) and the weight was measured. The water content of original cells and water content of cells in water/OE_2_imC_3_C (90/10, v/w) was calculated as following equations.

$${{{{{{{\mathrm{Water}}}}}}}}\;{{{{{{{\mathrm{content}}}}}}}}\;{{{{{{{\mathrm{of}}}}}}}}\;{{{{{{{\mathrm{oringinal}}}}}}}}\;{{{{{{{\mathrm{cells}}}}}}}}\;({{{{{{{\mathrm{\% }}}}}}}}) = \frac{{{{{{{{{\mathrm{Cell}}}}}}}}\;{{{{{{{\mathrm{weight}}}}}}}}\;{{{{{{{\mathrm{before}}}}}}}}\;{{{{{{{\mathrm{drying}}}}}}}} - {{{{{{{\mathrm{Cell}}}}}}}}\;{{{{{{{\mathrm{weight}}}}}}}}\;{{{{{{{\mathrm{after}}}}}}}}\;{{{{{{{\mathrm{drying}}}}}}}}}}{{{{{{{{{\mathrm{Cell}}}}}}}}\;{{{{{{{\mathrm{weight}}}}}}}}\;{{{{{{{\mathrm{before}}}}}}}}\;{{{{{{{\mathrm{drying}}}}}}}}}} \times 100$$

$${{{{{{{\mathrm{Water}}}}}}}}\;{{{{{{{\mathrm{content}}}}}}}}\;{{{{{{{\mathrm{of}}}}}}}}\;{{{{{{{\mathrm{cells}}}}}}}}\;{{{{{{{\mathrm{in}}}}}}}}\;{{{{{{{\mathrm{water/OE}}}}}}}}_2{{{{{{{\mathrm{imC}}}}}}}}_3{{{{{{{\mathrm{C}}}}}}}}\;(90/10,\;{{{{{{{\mathrm{v/w}}}}}}}})\;({{{{{{{\mathrm{\% }}}}}}}}) = \frac{{{{{{{{{\mathrm{Relative}}}}}}}}\;{{{{{{{\mathrm{cell}}}}}}}}\;{{{{{{{\mathrm{volume}}}}}}}} - (100 - {{{{{{{\mathrm{Water}}}}}}}}\;{{{{{{{\mathrm{content}}}}}}}}\;{{{{{{{\mathrm{of}}}}}}}}\;{{{{{{{\mathrm{original}}}}}}}}\;{{{{{{{\mathrm{cells}}}}}}}})}}{{{{{{{{{\mathrm{Relative}}}}}}}}\;{{{{{{{\mathrm{cell}}}}}}}}\;{{{{{{{\mathrm{volume}}}}}}}}}}$$

### Osmotic pressure of cryoprotectants

Osmotic pressure was measured using a vapor pressure osmometer (VAPRO 5600; Wescor, Inc., Logan, UT, USA) in the standard 10 mL chamber.

### Physical state of cryoprotectants under cryogenic temperature

The phase behavior of the solutions under cryogenic temperature was investigated using DSC (DSC-60A plus, Shimadzu Corporation). DSC measurements were conducted under the following conditions: cooling to −100 °C at a cooling rate of −1 °C/min followed by heating to 25 °C at a heating rate of 5 °C/min. The proportion of unfrozen water in the solutions was estimated from the area of the melting peak at ~0 °C and calculated using the following equation:

$${{{{{{{\mathrm{Proportion}}}}}}}}\;{{{{{{{\mathrm{of}}}}}}}}\;{{{{{{{\mathrm{unfrozen}}}}}}}}\;{{{{{{{\mathrm{water}}}}}}}}\;{{{{{{{\mathrm{in}}}}}}}}\;{{{{{{{\mathrm{solution}}}}}}}}\left( {{{{{{{\mathrm{\% }}}}}}}} \right) = \\ 100 - \frac{{{{{{{{{\mathrm{Melting}}}}}}}}\;{{{{{{{\mathrm{heat}}}}}}}}\;{{{{{{{\mathrm{of}}}}}}}}\;{{{{{{{\mathrm{the}}}}}}}}\;{{{{{{{\mathrm{sample}}}}}}}}\;{{{{{{{\mathrm{solutions}}}}}}}}\;[{{{{{{{\mathrm{J}}}}}}}}/{{{{{{{\mathrm{g}}}}}}}}]}}{{{{{{{{{\mathrm{Melting}}}}}}}}\;{{{{{{{\mathrm{heat}}}}}}}}\;{{{{{{{\mathrm{of}}}}}}}}\;{{{{{{{\mathrm{water}}}}}}}}\;\left( {265\;[{{{{{{{\mathrm{J}}}}}}}}/{{{{{{{\mathrm{g}}}}}}}}]} \right)\; \times {{{{{{{\mathrm{water}}}}}}}}\;{{{{{{{\mathrm{proportion}}}}}}}}\;{{{{{{{\mathrm{in}}}}}}}}\;{{{{{{{\mathrm{the}}}}}}}}\;{{{{{{{\mathrm{solution}}}}}}}}}} \times 100.$$

### Molecular dynamics simulation

Lipid bilayer contained 200 molecules of 1,2-dipalmitoyl-sn-glycero-3-phosphorylcholine (DPPC) was generated by PACKMOL-Memgen^28^. The cell membrane was placed at the center in a rectangular periodic box filled with the aqueous solution, where is water slabs with a thickness of 54 Å above and below the lipid bilayer. Simulation systems were constructed with two types of aqueous solution state, water/OE_2_imC_3_C/DMSO (10,000/94/462 molecules for 75/10/15, w/w/w) and water/DMSO (12,000/480 molecules for 85/15, w/w). Molecular dynamics simulations of the cell membrane systems were carried out using the SANDER and PMEMD.CUDA modules of the Amber18 and AmberTools19 software with the NVIDIA Pascal GPU system^29^.

The initial positions of water/OE_2_imC_3_C/DMSO (or water/DMSO) molecules were optimized by 15,000 cycles of steepest descent energy minimization, followed by 10,000 cycles of conjugated gradient minimization, while the structures of the lipid molecules were fixed with the constrained force of 500 kcal/(mol A^2^). The whole systems were then subjected to a combination of 15,000 cycles of steepest descent and 10,000 cycles of conjugated gradient energy minimizations. In the subsequent dynamics calculations, the cell membrane system was equilibrated by NVT ensemble simulations with a gradual increase in temperature from −253 to −153 °C at a rate of 0.1 °C/ps, followed by NPT ensemble simulations with a gradual increase in temperature from −153 to 37 °C at a rate of 0.1 °C/ps and pressure of 1 bar. Throughout the heating process, the motion of the lipid molecules was fixed by imposing positional constraints with a constrained force of 10 kcal/(mol A^2^). In addition, the NPT ensemble simulations to equilibrate the density of the system were performed for a total of 5 ns by repeating ten times at constant temperature (37 °C) and pressure (1 bar). Finally, the production simulations were carried out for 1 µs at constant temperature (37 °C) and pressure (1 bar).

The TIP3P model was used for the water molecules, and the lipid molecules were described by the Lipid17 force field^30^. DMSO and OE_2_imC_3_C molecules were modeled using the general AMBER force field^31^. The temperature and pressure of the system were regulated by a Langevin thermostat with a collision frequency of 1 ps^−1^ and a Berendsen barostat, respectively. The molecular dynamics simulation was performed using a 2 fs integration time step coupled with the SHAKE option. The particle mesh Ewald method was adopted for long-range interactions, and the cutoff for non-bonding interactions in the coordinate space was fixed at 10 Å.

### Reporting summary

Further information on research design is available in the Nature Research Reporting Summary linked to this article.

## Supplementary information

Supplementary Materials

Reporting Summary

## Data Availability

All data are available in the main text or supplementary materials.
