# Supplementary material for: Synthetic zwitterions as efficient non-permeable cryoprotectants
Source: Commun Chem. 2021 Oct 28;4:151. doi: 10.1038/s42004-021-00588-x (PMC9814846; doi:10.1038/s42004-021-00588-x)
Supplement: Supplementary file 1 — Supplementary Materials [file 42004_2021_588_MOESM1_ESM.pdf]

Supporting information

Synthetic zwitterions as efficient non-permeable cryoprotectants

Yui Kato, Takuya Uto, Daisuke Tanaka, Kojiro Ishibashi, Akiko Kobayashi, Masaharu Hazawa, Richard W. Wong, Kazuaki Ninomiya, Kenji Takahashi, Eishu Hirata*, Kosuke Kuroda*

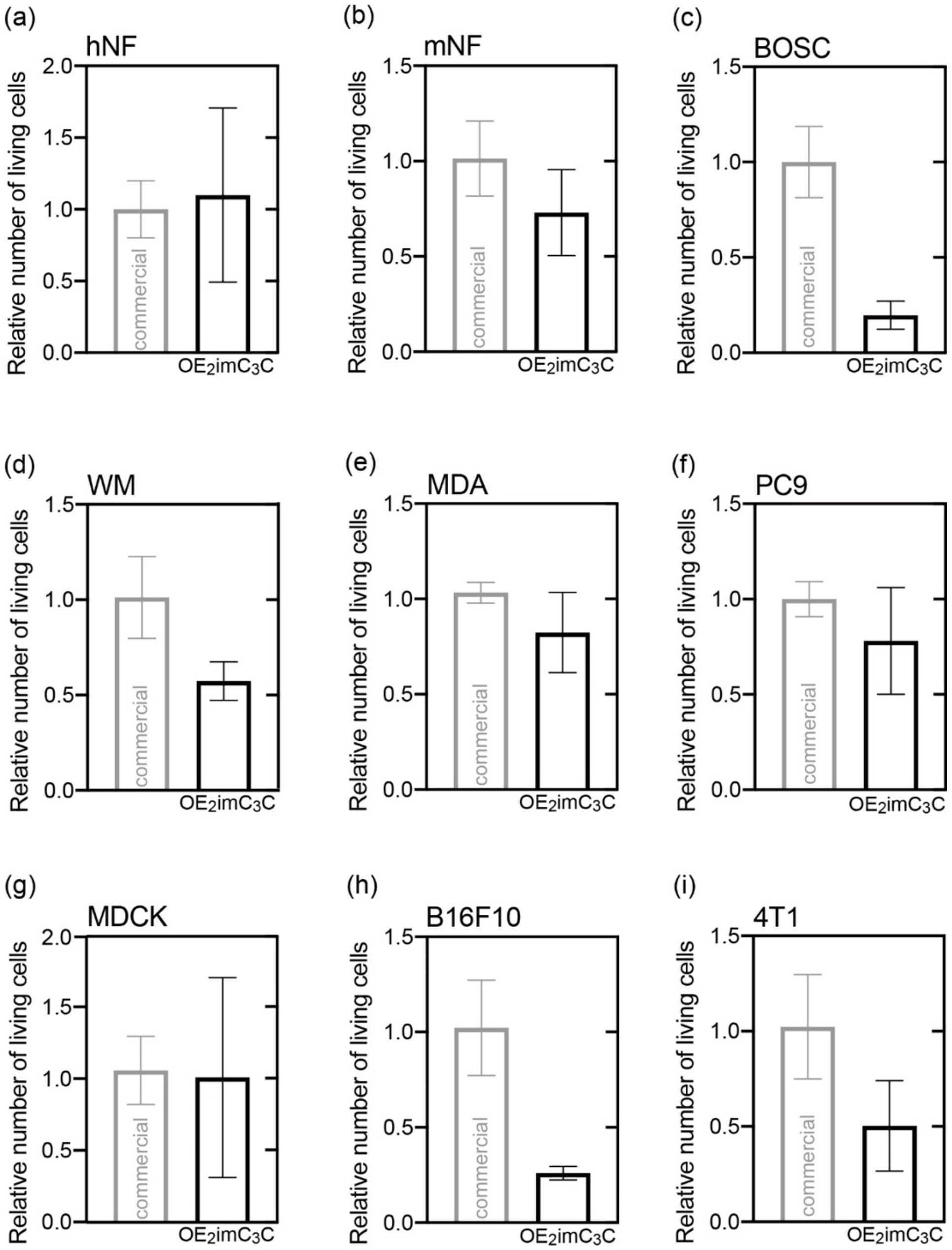

Figure S1 Relative number of living cells after cryopreservation using a commercial cryoprotectant and water/OE_2imC_3C (95/5, v/w). (n = 3, biologically triplicate) The bars show standard error. These cell numbers were counted by ®Countess II FL. Commercial cryoprotectant is Culture Sure freezing medium (Fujifilm Wako Pure Chemical Corporation).

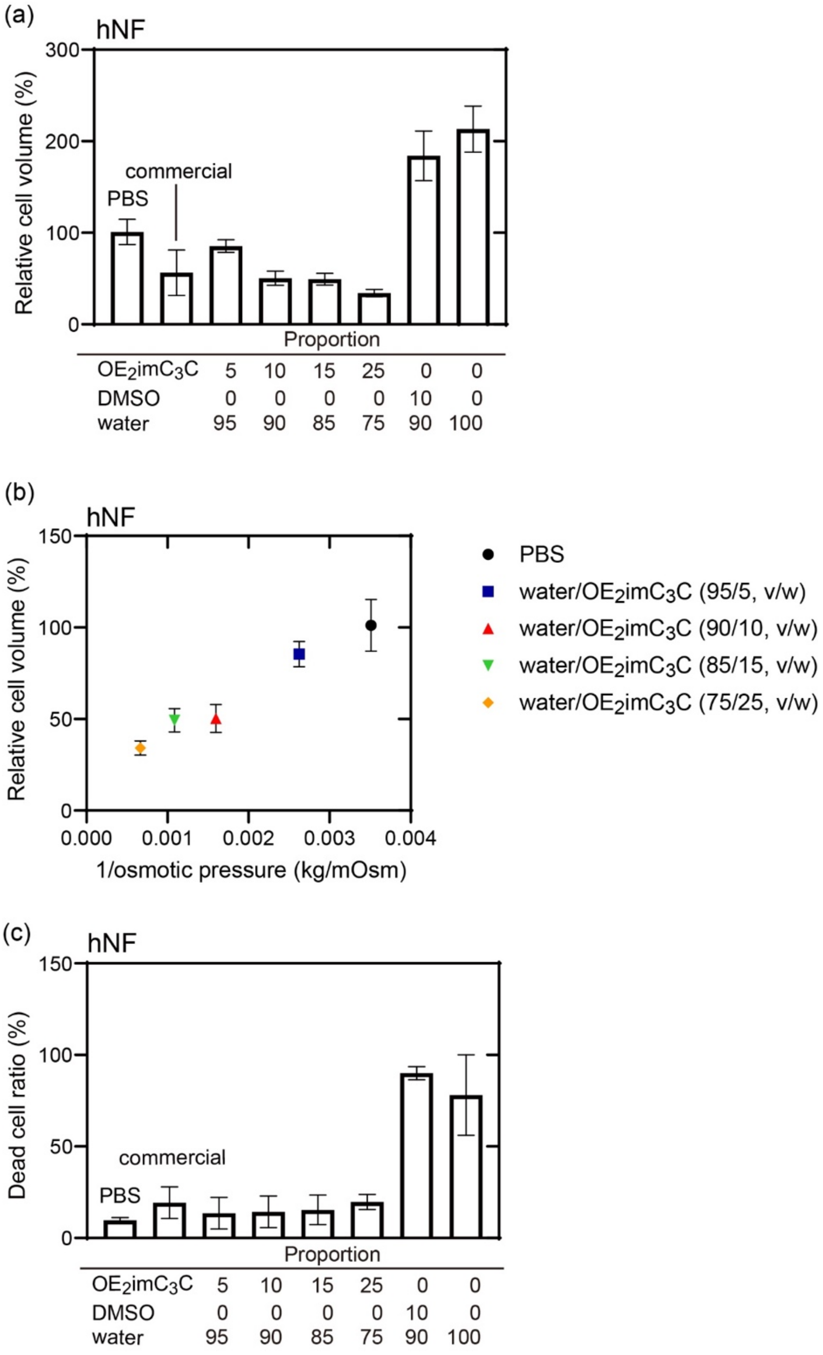

Figure S2 (a) Relative cell volumes of hNF in the indicated concentration after 5 min immersion. (n = 3, biologically triplicate) The hNF was immersed as floating cells in the OE_2imC_3C solutions after trypsinization. (b) Relative hNF cell volumes in the indicated OE_2imC_3C solutions and PBS (standardized as 100%) after 5 min immersion. The plots correspond to PBS and 5, 10, 15, and 25% (w/v) OE_2imC_3C solutions (from right to left). (c) Dead hNF cell ratio in the indicated solutions after 60 min immersion. (n = 3, biologically triplicate) The hNF was immersed as floating cells in the solutions after trypsinization. Commercial cryoprotectant is Culture Sure freezing medium (Fujifilm Wako Pure Chemical Corporation). All experiments were performed at room temperature. The bars show standard error.

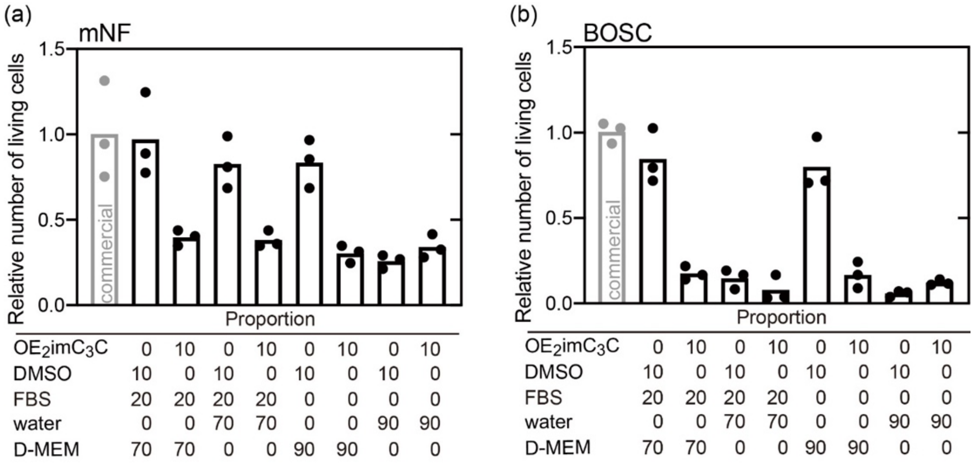

Figure S3 Relative number of living (a) mNF and (b) BOSC cells after cryopreservation with the indicated freezing media. (n = 3, experimentally triplicate) In this experiment, DMSO was measured in volume. Commercial cryoprotectant is Culture Sure freezing medium (Fujifilm Wako Pure Chemical Corporation).

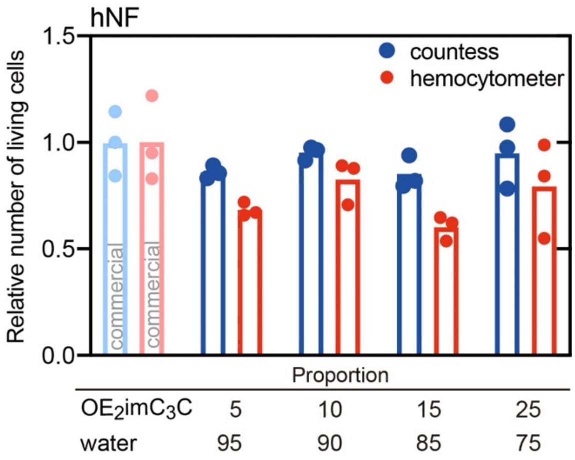

Figure S4 Relative number of living cells of hNF after cryopreservation counted by using ®Countess II FL and hemocytometers. The cells were taken from the same samples and subjected to counting by both methods. Commercial cryoprotectant is Culture Sure freezing medium (Fujifilm Wako Pure Chemical Corporation). (n = 3, experimentally triplicate)

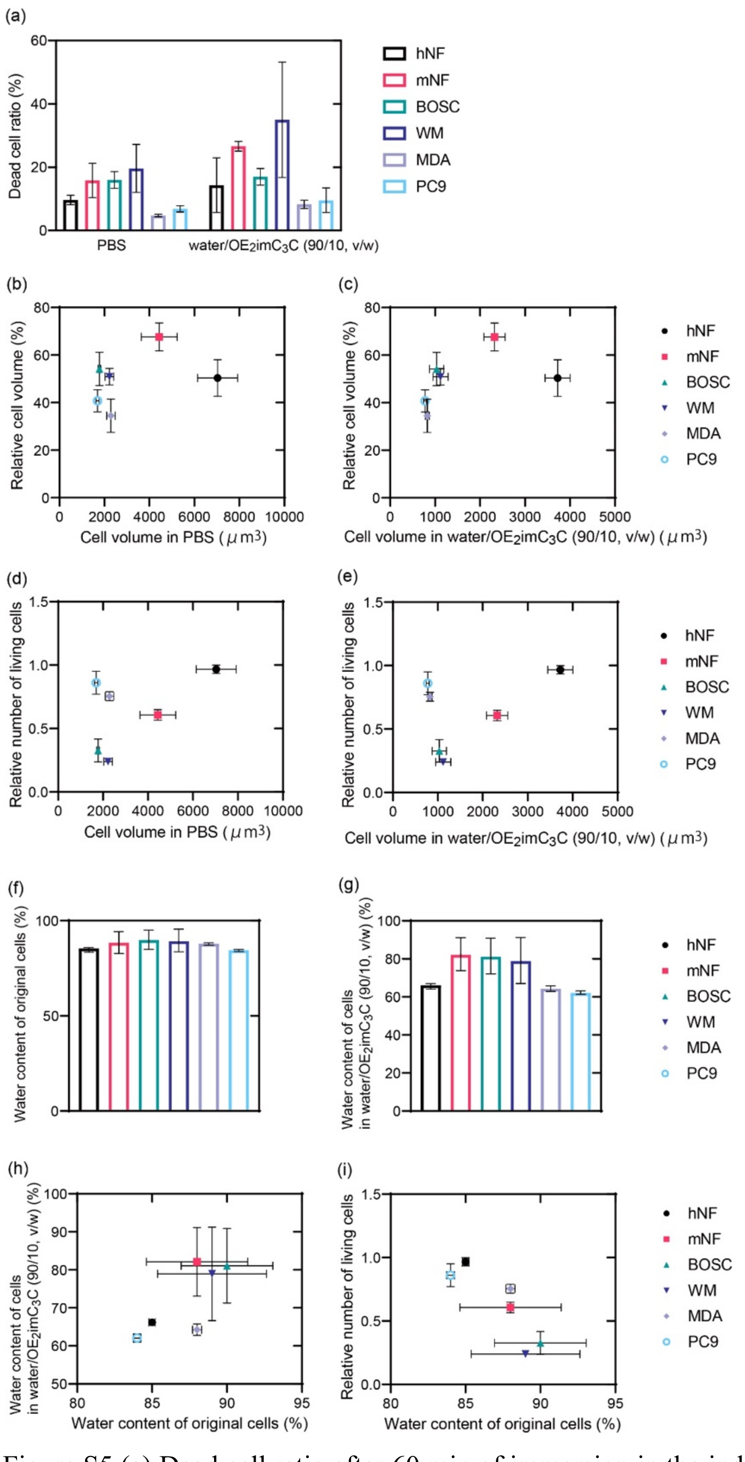

Figure S5 (a) Dead cell ratio after 60 min of immersion in the indicated solution (n = 3, biologically triplicate). For (a), these cells were immersed as floating cells in the solutions after trypsinization. Relation between relative cell volume after 5 min of immersion in water/OE_2imC_3C (90/10, v/w) and absolute cell volume in (b) PBS and (c) water/OE_2imC_3C (90/10, v/w) after 5 min of immersion. Relation between relative number of living cells and absolute cell volume in (d) PBS and (e) water/OE_2imC_3C (90/10, v/w) after 5 min of immersion. Water content of (f) original cells in PBS and (g) cells dehydrated in water/OE_2imC_3C (90/10, v/w). (h) Relation between water content of original cells and cells dehydrated in water/OE_2imC_3C (90/10, v/w). (i) Relation between relative number of living cells and water content of original cells. All experiments were performed at room temperature. The bars show standard error.

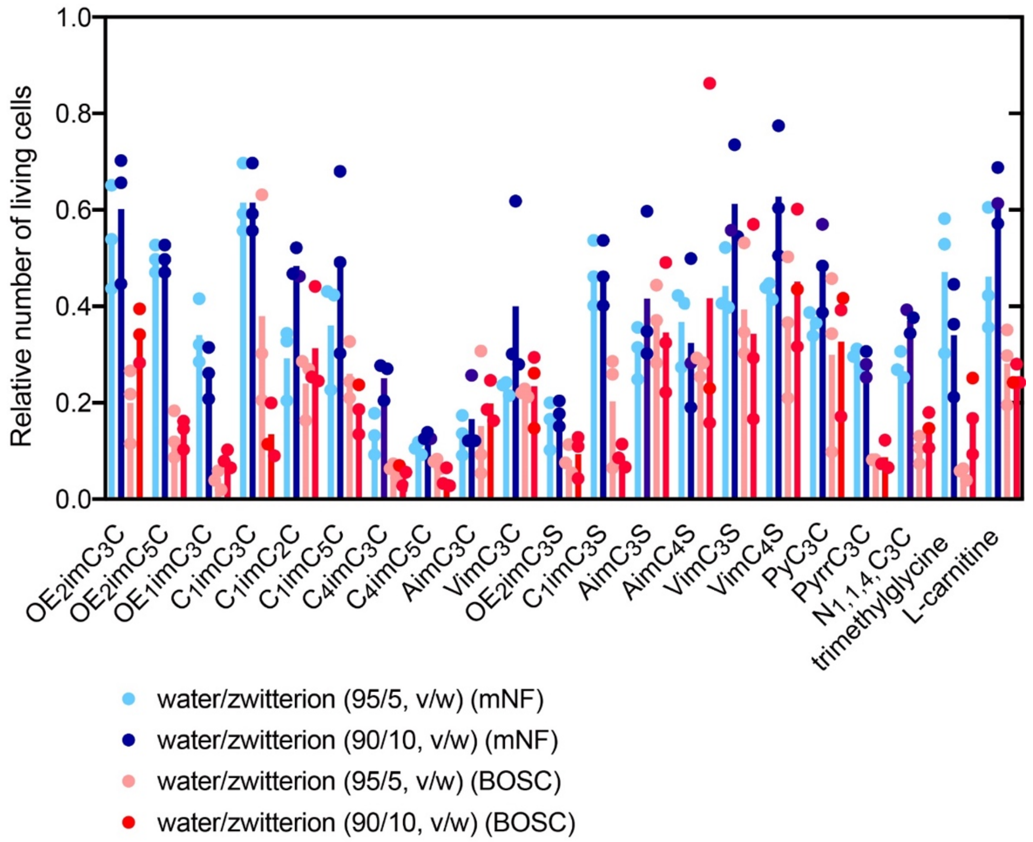

Figure S6 Relative number of living mNF and BOSC cells after cryopreservation using 5% and 10% (w/v) zwitterion solutions. (n = 3, experimentally triplicate) This figure is basically reproduced from Figure 4.

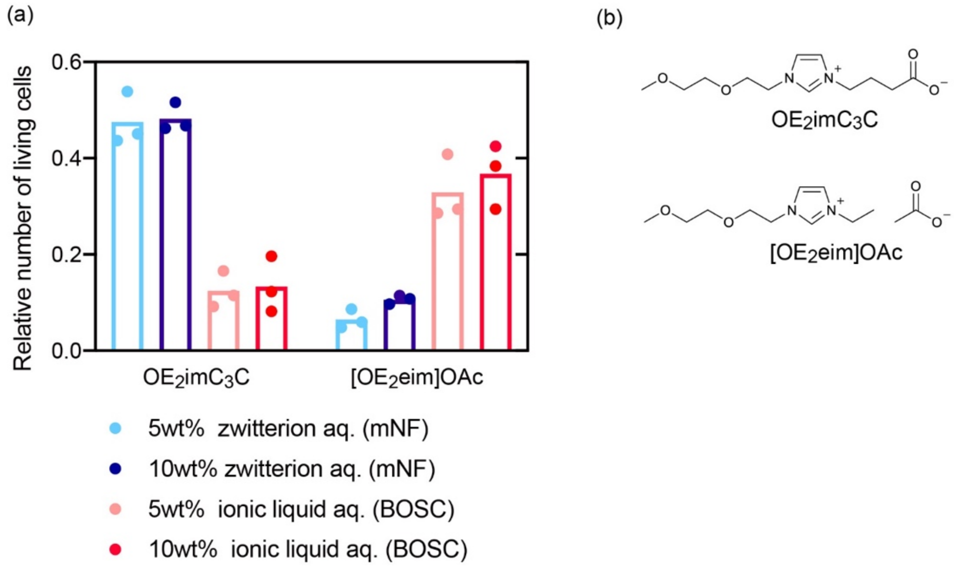

Figure S7 (a) Relative number of living mNF and BOSC cells after cryopreservation using 5% and 10% (w/v) $[OE_2eim]OAc$ and OE_2imC_3C solutions. (n = 3, experimentally triplicate) (b) Structures of OE_2imC_3C and $[OE_2eim]OAc$.

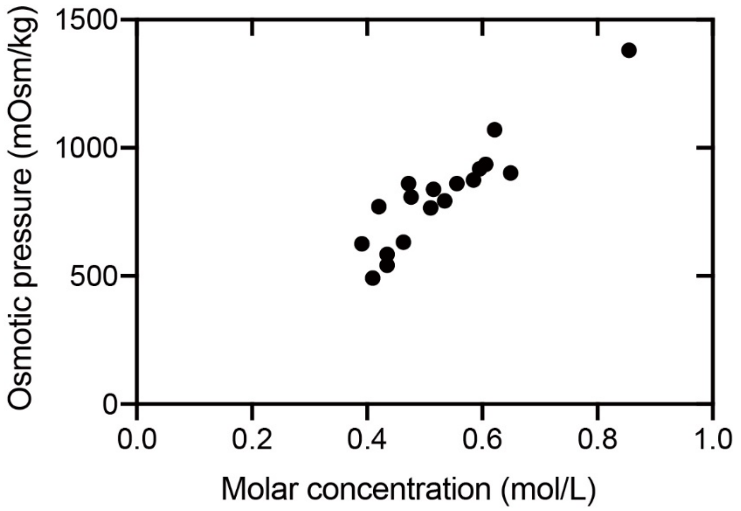

Figure S8 Relationship between osmotic pressure and molar concentration of water/zwitterion (90/10, v/w) solutions.

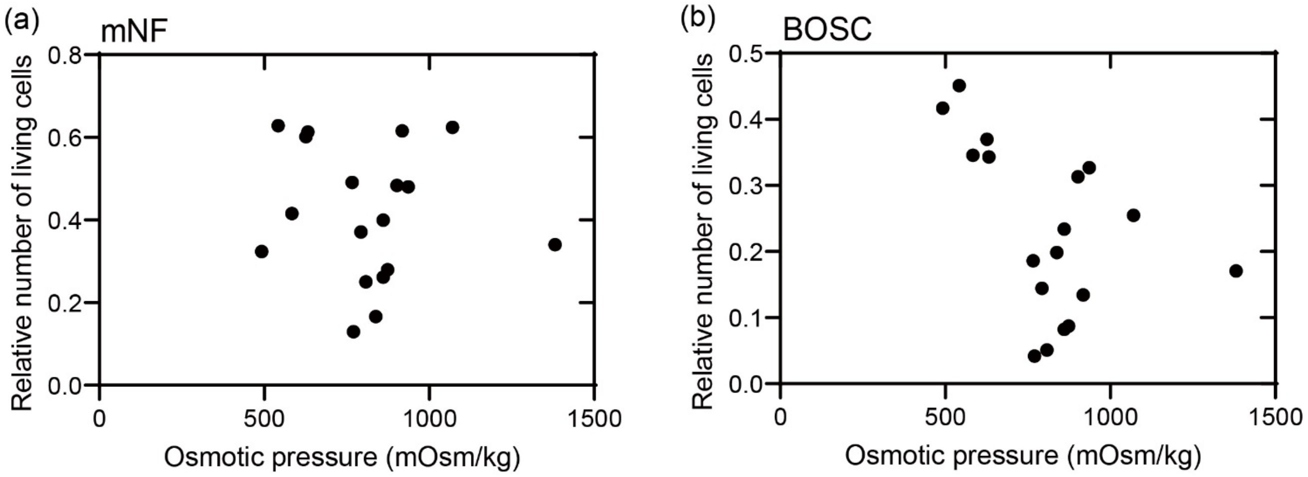

Figure S9 Relationship between osmotic pressure of water/zwitterion (90/10, v/w) and relative number of living (a) mNF and (b) BOSC cells after cryopreservation in the solutions.

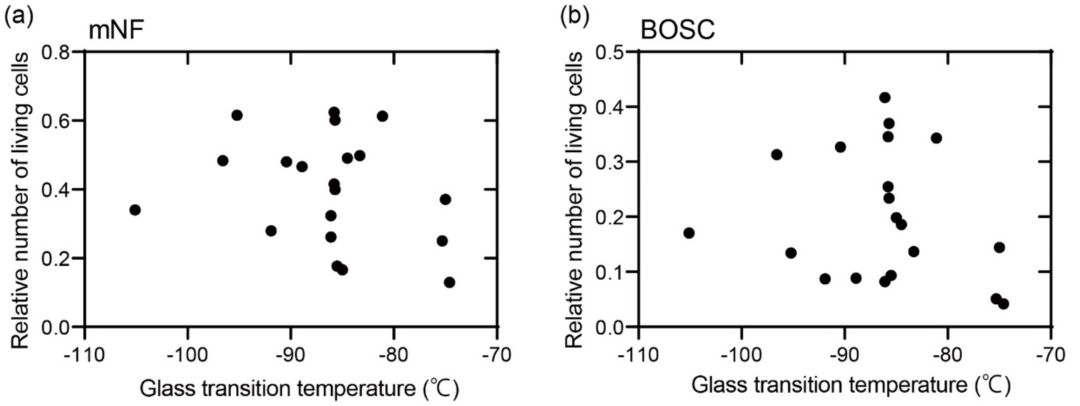

Figure S10 Relation between glass transition temperature of water/zwitterion (90/10, v/w) and relative number of living (a) mNF and (b) BOSC cells after cryopreservation in the solutions.

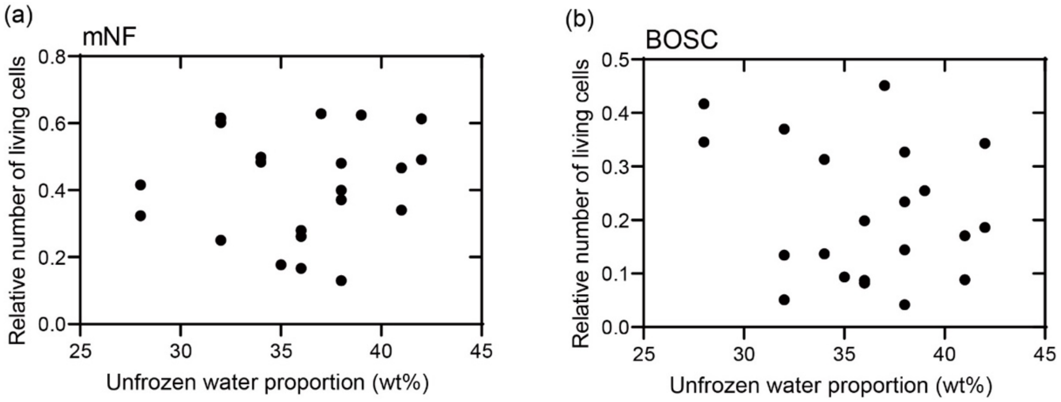

Figure S11 Relation between unfrozen water proportion of water/zwitterion (90/10, v/w) and relative number of living (a) mNF and (b) BOSC cells after cryopreservation in the solutions.

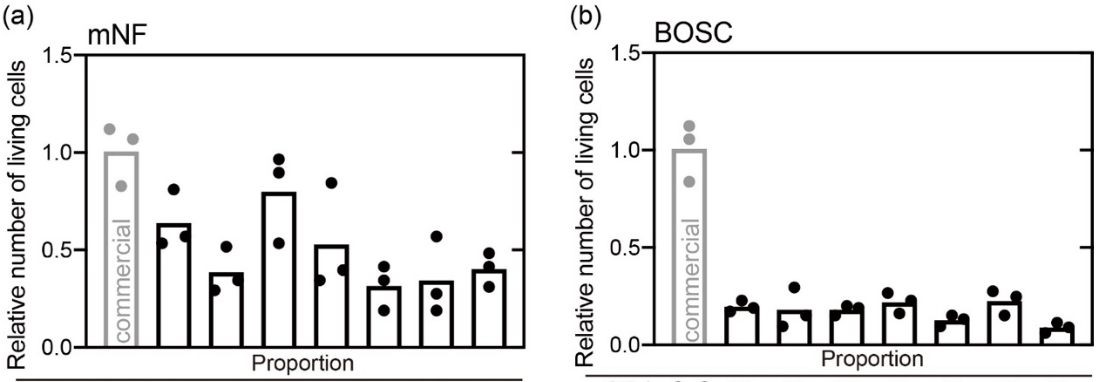

(a)

OE_2imC_3C	10	10	10	10	10	10	10
C_1imC_3C	0	10	0	0	0	0	0
L-carnitine	0	0	10	0	0	0	0
$AimC_3C$	0	0	0	10	0	0	0
$AimC_3S$	0	0	0	0	10	0	0
$AimC_4S$	0	0	0	0	0	10	0
$VimC_4S$	0	0	0	0	0	0	10
water	80	80	80	80	80	80	80

(b)

OE_2imC_3C	10	10	10	10	10	10	10
C_1imC_3C	0	10	0	0	0	0	0
L-carnitine	0	0	10	0	0	0	0
$AimC_3C$	0	0	0	10	0	0	0
$AimC_3S$	0	0	0	0	10	0	0
$AimC_4S$	0	0	0	0	0	10	0
$VimC_4S$	0	0	0	0	0	0	10
water	80	80	80	80	80	80	80

Figure S12 Relative number of living (a) mNF and (b) BOSC cells after cryopreservation with water/zwitterion/zwitterion (80/10/10, v/w/w). (n = 3, experimentally triplicate) Commercial cryoprotectant is Culture Sure freezing medium (Fujifilm Wako Pure Chemical Corporation).

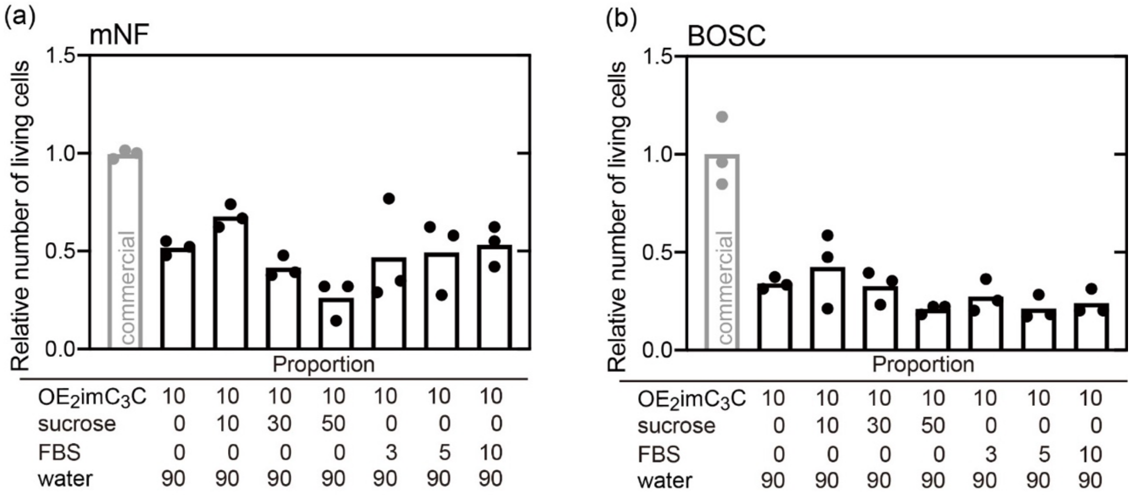

Figure S13 Relative number of living (a) mNF and (b) BOSC cells after cryopreservation with water/OE_2imC_3C (90/10, v/w) and non-cell-permeable additives (sucrose and FBS). (n = 3, experimentally triplicate) Commercial cryoprotectant is Culture Sure freezing medium (Fujifilm Wako Pure Chemical Corporation).

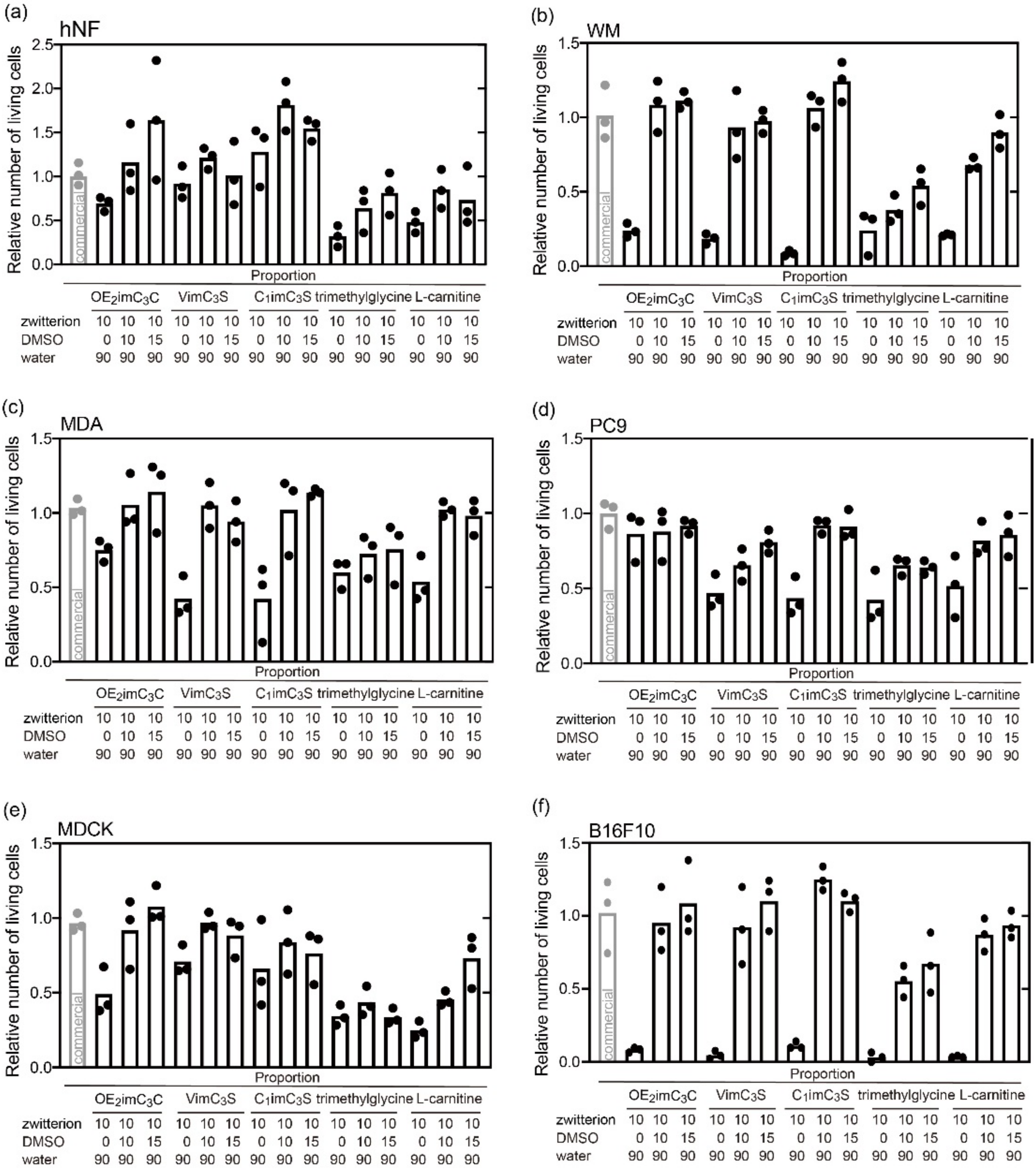
(a)
hNF
(b)
WM
(c)
MDA
(d)
PC9
(e)
MDCK
(f)
B16F10
Relative number of living cells
commercial
Proportion
OE$_2$imC$_3$C
VimC$_3$S
C$_1$imC$_3$S
trimethylglycine
L-carnitine
zwitterion 10 10 10 10 10 10 10 10 10 10 10 10 10 10 10
DMSO 0 10 15 0 10 15 0 10 15 0 10 15 0 10 15
water 90 90 90 90 90 90 90 90 90 90 90 90 90 90 90

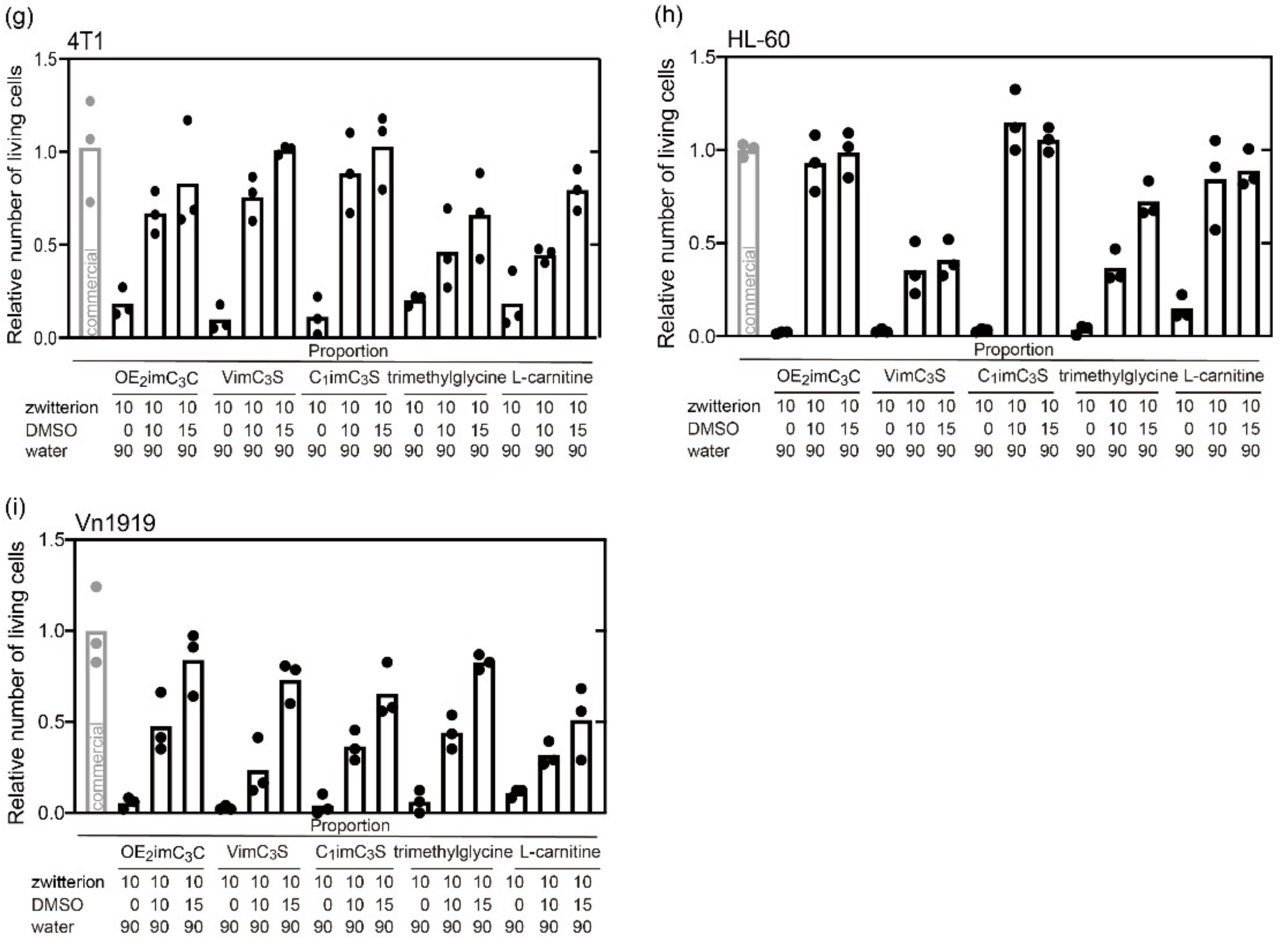

Figure S14 Relative number of living (a) hNF, (b) WM, (c) MDA, (d) PC9, (e) MDCK, (f) B16F10, (g) 4T1, (h) HL-60 and (i) Vn1919 cells after cryopreservation with water/zwitterion (90/10, v/w) and DMSO. (n = 3, experimentally triplicate) Commercial cryoprotectant is Culture Sure freezing medium (Fujifilm Wako Pure Chemical Corporation).

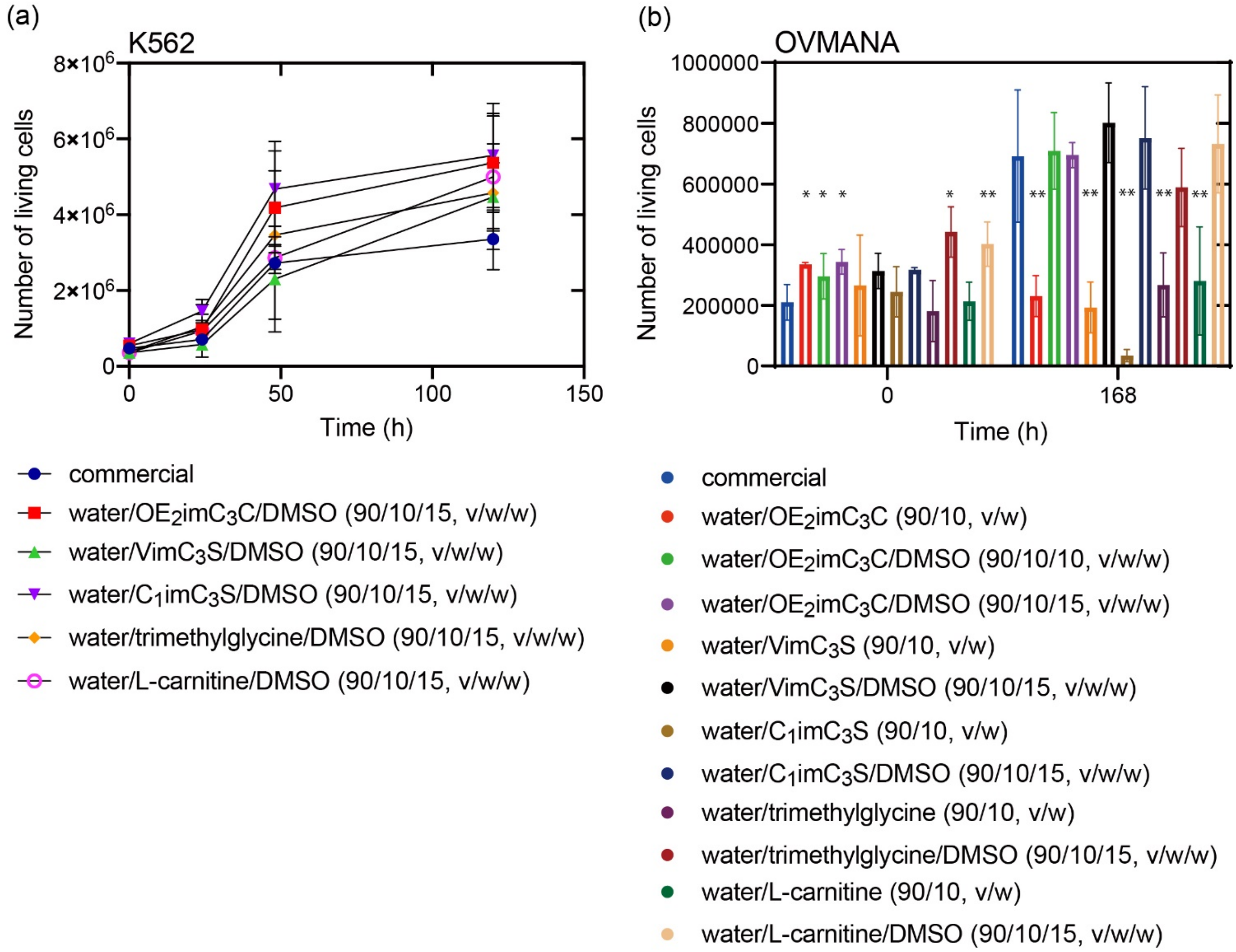

Figure S15 (a) Growth curves of K562 cells after cryopreservation with the indicated solutions. (n = 3, experimentally triplicate) (b) Number of living cells of OVMANA at 0 and 336 h after freezing and thawing with the indicated solutions (*: $p<0.1$, **: $p<0.05$). (n = 3, experimentally triplicate) Commercial cryoprotectant is Culture Sure freezing medium (Fujifilm Wako Pure Chemical Corporation). The bars show standard error.

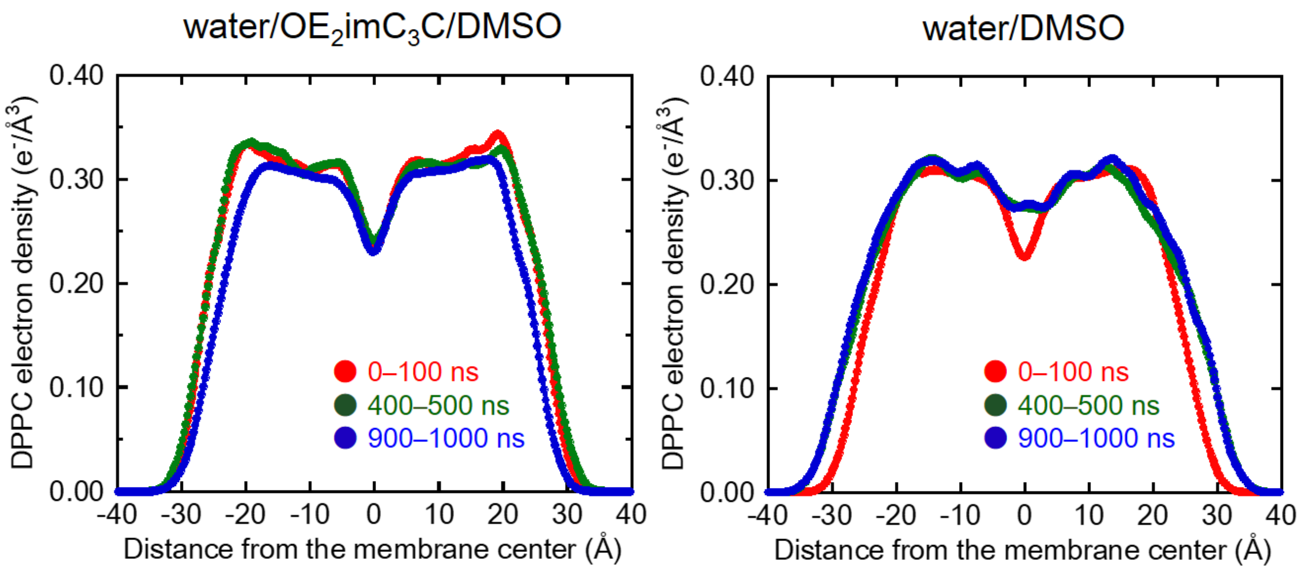

Figure S16 Electron density profiles of DPPC lipid molecules in the water/OE$_2$imC$_3$C/DMSO (left) and water/DMSO (right) solutions calculated using molecular dynamics (MD) trajectories.

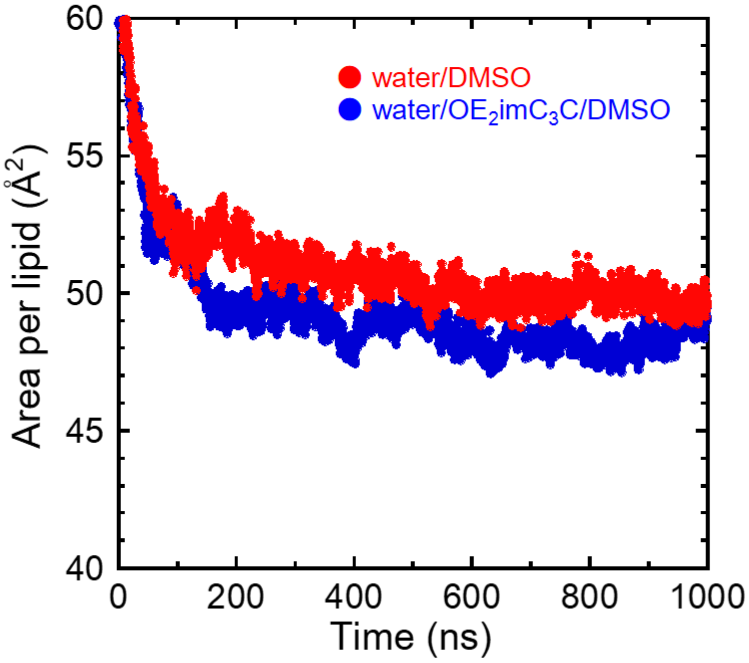

Figure S17 Time series of area per lipid of cell membrane in the water/DMSO (red) and water/OE$_2$imC$_3$C/DMSO (blue) solutions calculated using MD trajectories.

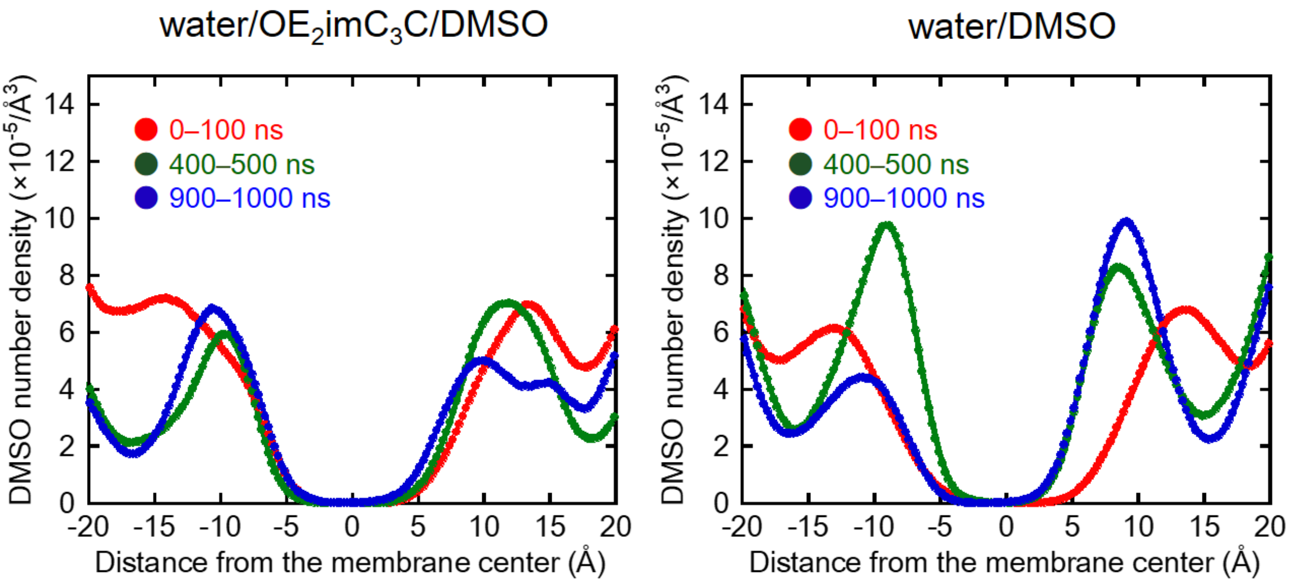

Figure S18 Number density of DMSO around the cell membrane center in the water/OE_2imC_3C/DMSO (left) and water/DMSO (right) calculated using MD trajectories.

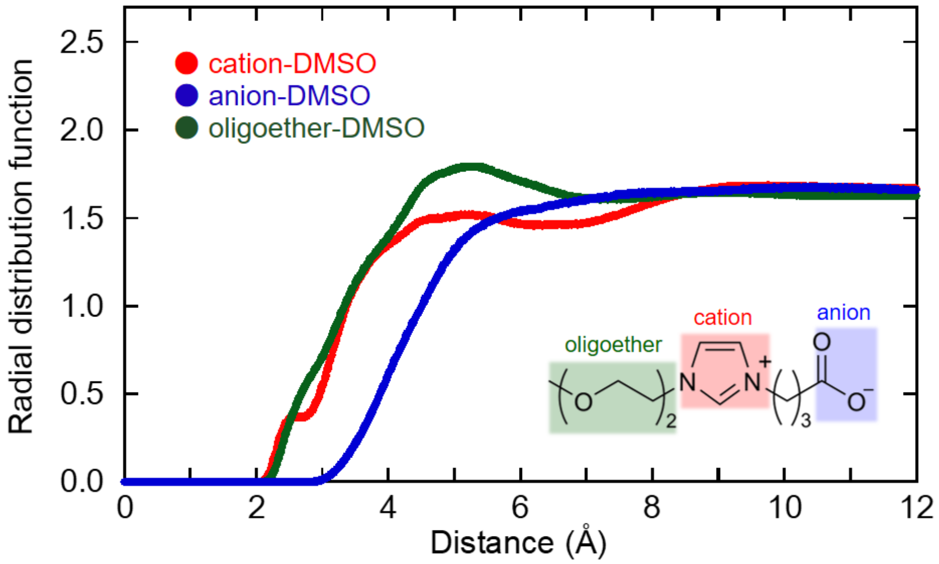

Figure S19 Radial distribution functions involving the highlighted part of OE_2imC_3C-DMSO in the water/OE_2imC_3C/DMSO solution from MD trajectories for 1 μs.

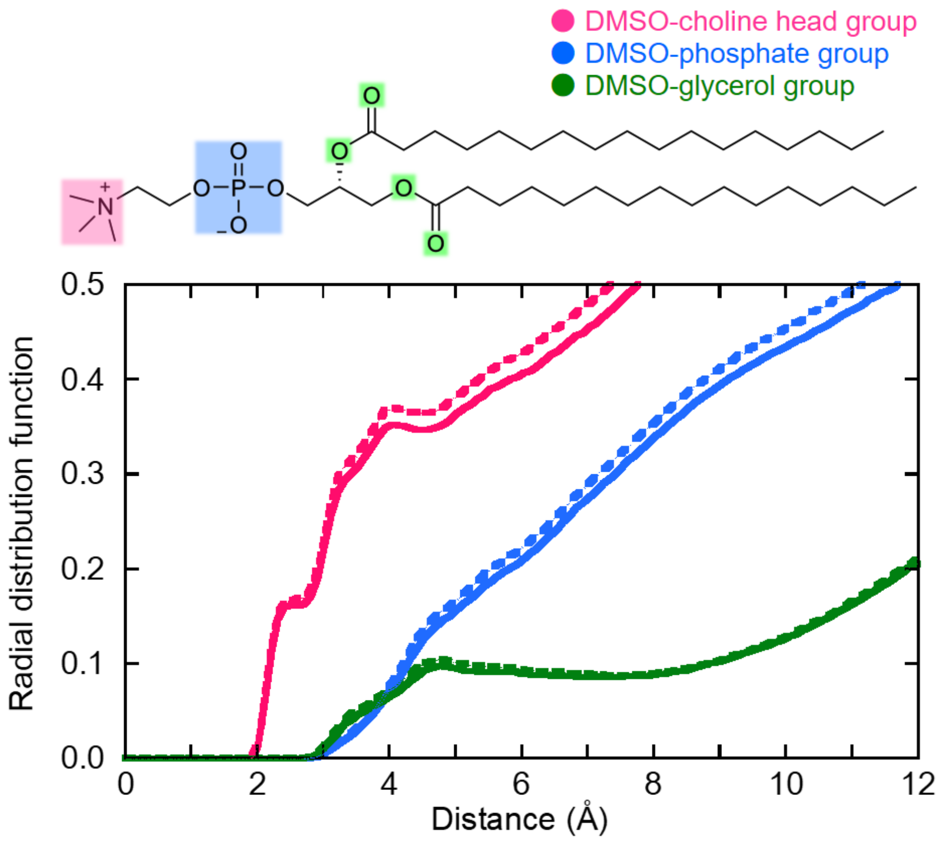

Figure S20 Radial distribution functions involving the highlighted part of DPPC-DMSO in the water/DMSO (dotted lines) and the water/OE_2imC_3C/DMSO (solid lines) from MD trajectories for 1 μs.

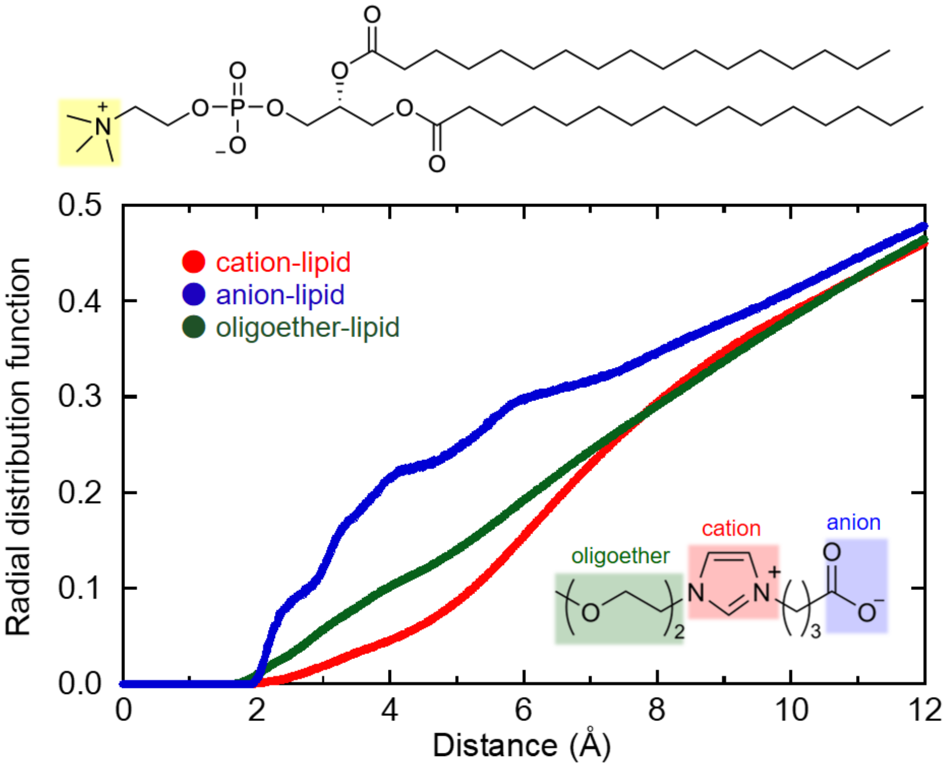

Figure S21 Radial distribution functions involving the highlighted part of OE_2imC_3C-DPPC (highlighted choline head group) in the water/OE_2imC_3C/DMSO solution from MD trajectories for 1 μs.

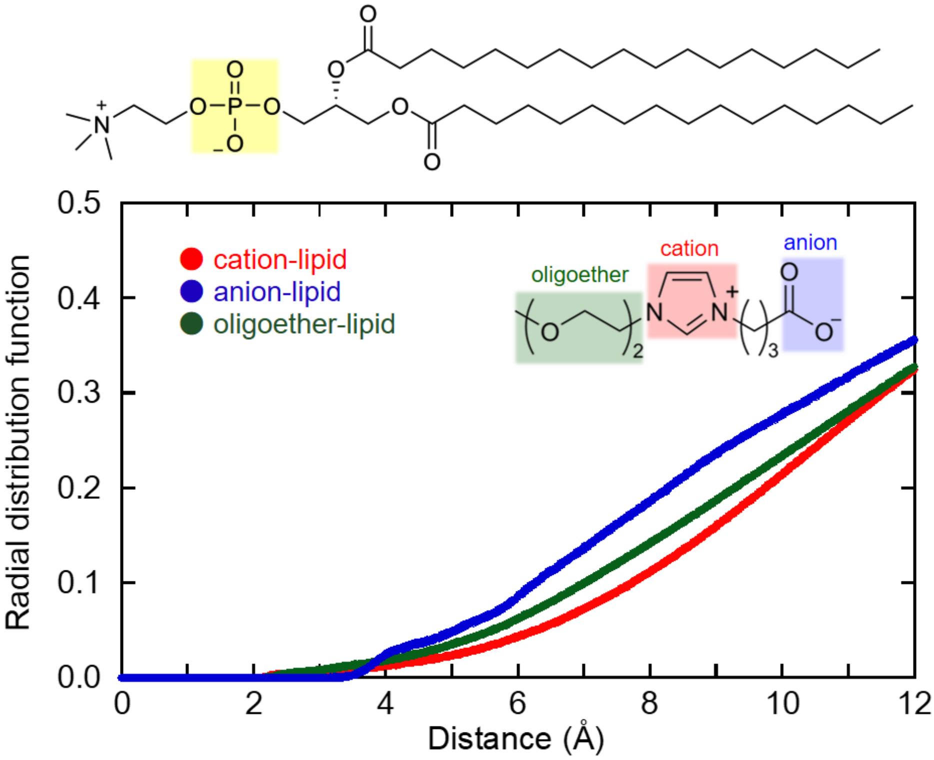

Figure S22 Radial distribution functions involving the highlighted part of OE_2imC_3C-DPPC (highlighted phosphate group) in the water/OE_2imC_3C/DMSO solution from MD trajectories for 1 μs.

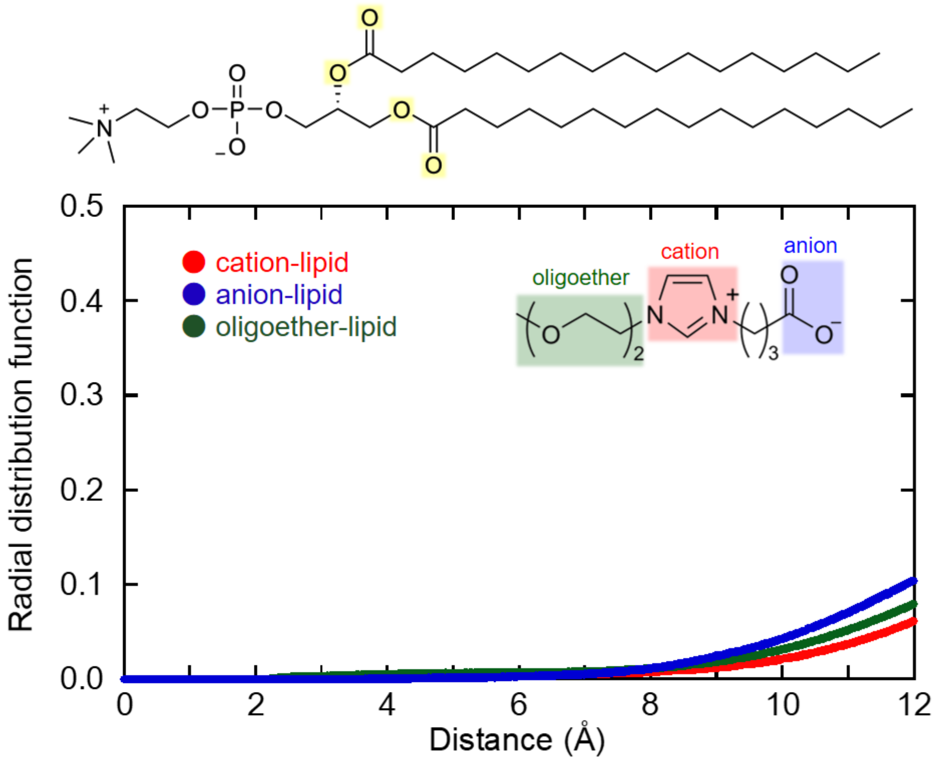

Figure S23 Radial distribution functions involving the highlighted part of OE_2imC_3C-DPPC (highlighted glycerol group) in the water/OE_2imC_3C/DMSO solution from MD trajectories for 1 μs.

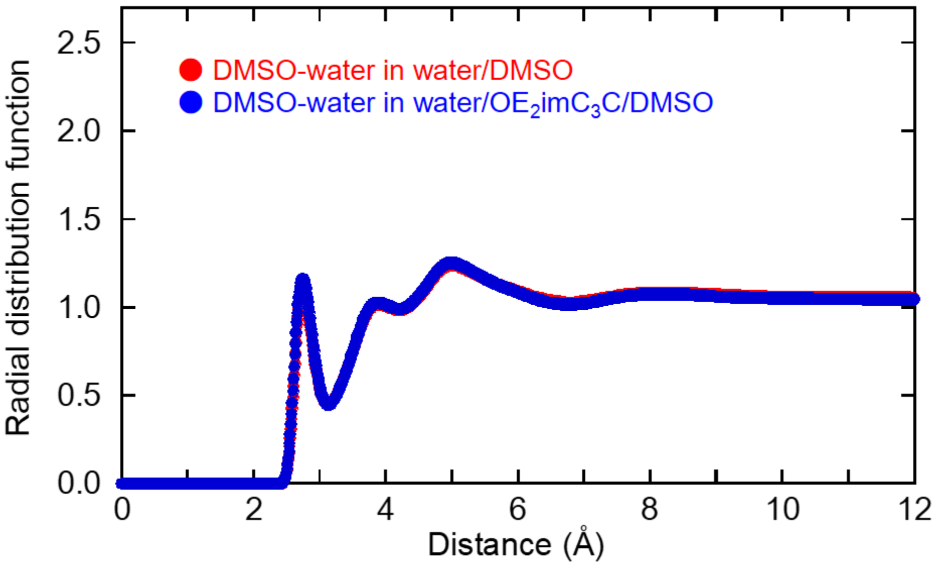

Figure S24 Radial distribution functions involving the highlighted DMSO-water in the water/DMSO (red) and the water/OE_2imC_3C/DMSO (blue) from MD trajectories for 1 μs.

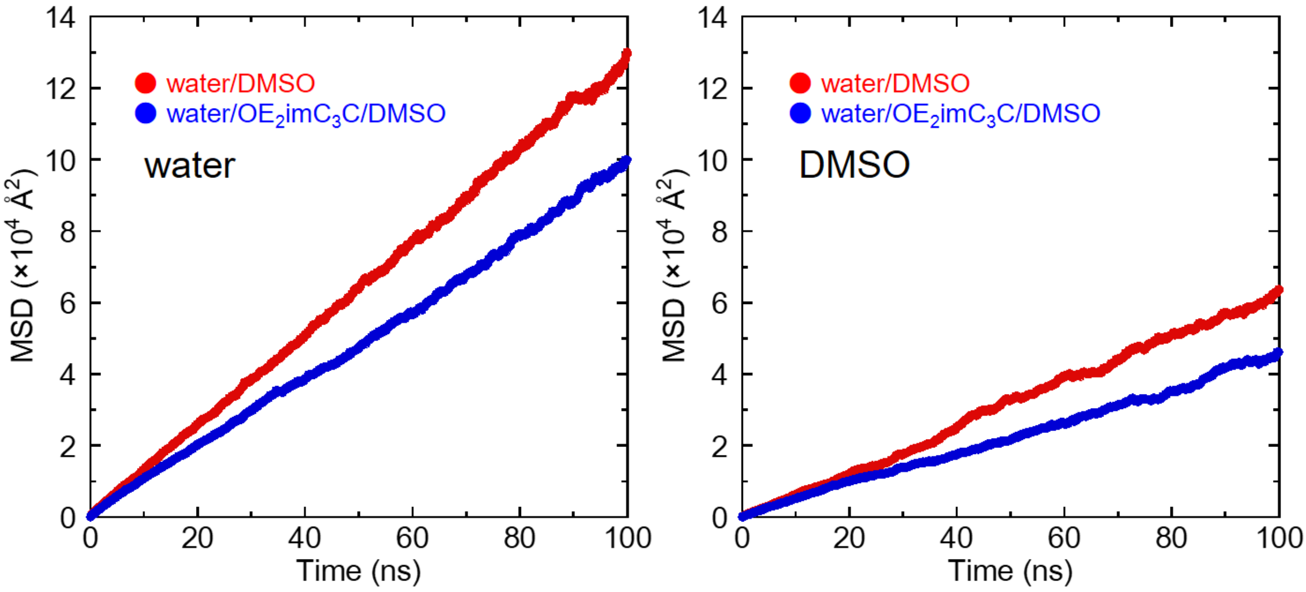

Figure S25 Mean square displacements (MSDs) of all water and DMSO molecules in the water/DMSO (red) and water/OE_2imC_3C/DMSO (blue) calculated using MD trajectories for 100 ns after equilibration. The diffusion constants were calculated by fitting slope to MSD vs time for 100 ns. The diffusion coefficients of water were 1.06×10^{-3} and 0.81×10^{-3} cm^2/s in the water/DMSO and the water/OE_2imC_3C/DMSO solutions, respectively. On the other hand, the diffusion coefficients of DMSO were 0.53×10^{-3} and 0.37×10^{-3} cm^2/s in the water/DMSO and the water/OE_2imC_3C/DMSO solutions, respectively.

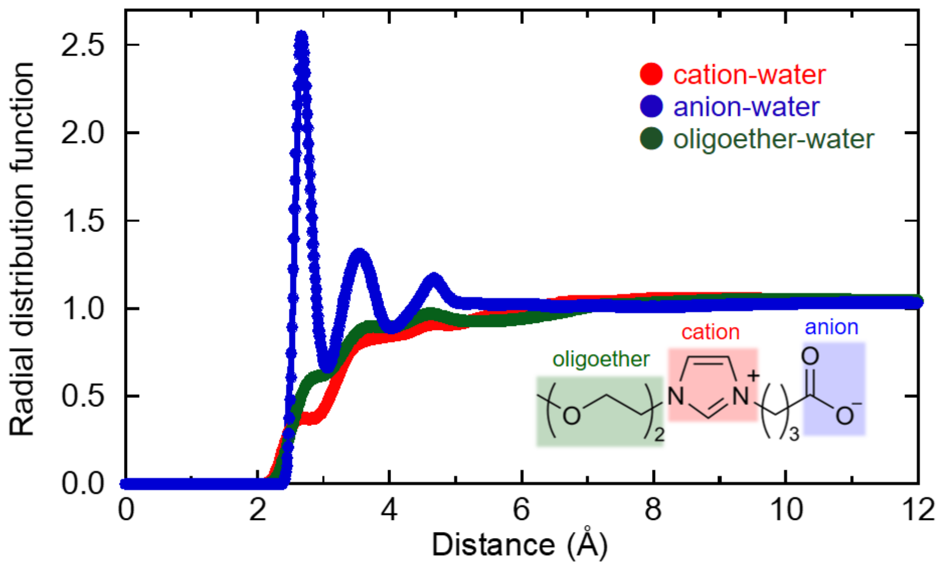

Figure S26 Radial distribution functions involving the highlighted part of OE_2imC_3C-water in the water/OE_2imC_3C/DMSO solution from MD trajectories for 1 μs.

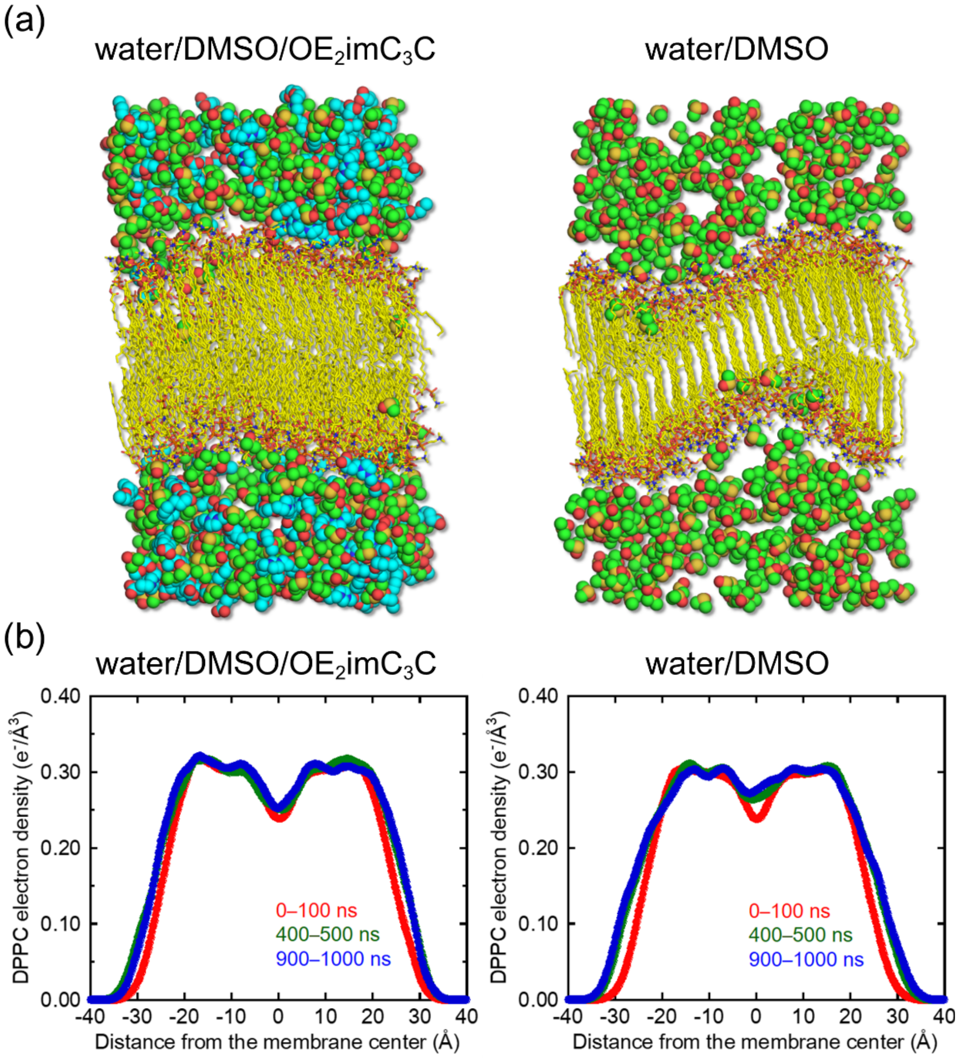

Figure S27 MD simulation data of the cell membrane system calculated with Lipid14 force field. (a) MD snapshot at 1,000 ns, (b) Electron density profiles of DPPC lipid molecules in the water/OE_2imC_3C/DMSO (left) and water/DMSO (right) solutions calculated using MD trajectories.

We here confirmed the reproducibility of the MD simulation calculated with Lipid17. Figure S27 shows the MD structures and the electron density profiles of DPPC lipid molecules calculated with Lipid14 force field. Despite a certain degree of disruption, the cell membrane did not critically collapse in the water/OE_2imC_3C/DMSO, while the lipid membrane significantly changed in water/DMSO. In this respect, the observed trends were similar to the cases calculated with the Lipid17 although they are not completely the same.

Table S1 Glass transition temperature and unfrozen water proportion of the solutions of water/zwitterion (90/10, v/w), and relative number of living mNF and BOSC cells after cryopreservation in the indicated solutions.

	Glass transition temperature T_g (°C)	Unfrozen water proportion (wt%)	Relative number of living cells	
			mNF	BOSC
OE_2imC_3C	-86	17	0.60	0.37
OE_2imC_5C	-83	19	0.50	0.14
OE_1imC_3C	-86	21	0.26	0.08
C_1imC_3C	-95	16	0.62	0.13
C_1imC_2C	-97	18	0.48	0.31
C_1imC_5C	-85	29	0.49	0.19
C_4imC_3C	-75	17	0.25	0.05
C_4imC_5C	-75	23	0.13	0.04
$AimC_3C$	-85	21	0.17	0.20
$VimC_3C$	-86	24	0.40	0.23
OE_2imC_3S	-85	20	0.18	0.09
C_1imC_3S	-89	27	0.47	0.09
$AimC_3S$	-86	11	0.42	0.35
$AimC_4S$	-86	12	0.32	0.42
$VimC_3S$	-81	28	0.61	0.34
$VimC_4S$	-[a]	10	0.63	0.45
PyC_3C	-90	23	0.48	0.33
$PyrrC_3C$	-92	21	0.28	0.09
$N_{1,1,4,}C_3C$	-75	23	0.37	0.14
trimethylglycine	-105	27	0.34	0.17
L-carnitine	-86	25	0.62	0.25

[a]not detected over -100 °C
